# Upgrade of a Scanning Confocal Microscope to a Single-Beam Path STED Microscope

**DOI:** 10.1371/journal.pone.0130717

**Published:** 2015-06-19

**Authors:** André Klauss, Marcelle König, Carsten Hille

**Affiliations:** 1 Department of Physical Chemistry/ Applied Laser Sensing in Complex Biosystems (ALS ComBi), Institute of Chemistry, University of Potsdam, Potsdam, Germany; 2 PicoQuant GmbH, Berlin, Germany; Pennsylvania State Hershey College of Medicine, UNITED STATES

## Abstract

By overcoming the diffraction limit in light microscopy, super-resolution techniques, such as stimulated emission depletion (STED) microscopy, are experiencing an increasing impact on life sciences. High costs and technically demanding setups, however, may still hinder a wider distribution of this innovation in biomedical research laboratories. As far-field microscopy is the most widely employed microscopy modality in the life sciences, upgrading already existing systems seems to be an attractive option for achieving diffraction-unlimited fluorescence microscopy in a cost-effective manner. Here, we demonstrate the successful upgrade of a commercial time-resolved confocal fluorescence microscope to an easy-to-align STED microscope in the single-beam path layout, previously proposed as “easy-STED”, achieving lateral resolution < λ/10 corresponding to a five-fold improvement over a confocal modality. For this purpose, both the excitation and depletion laser beams pass through a commercially available segmented phase plate that creates the STED-doughnut light distribution in the focal plane, while leaving the excitation beam unaltered when implemented into the joint beam path. Diffraction-unlimited imaging of 20 nm-sized fluorescent beads as reference were achieved with the wavelength combination of 635 nm excitation and 766 nm depletion. To evaluate the STED performance in biological systems, we compared the popular phalloidin-coupled fluorescent dyes Atto647N and Abberior STAR635 by labeling F-actin filaments *in vitro* as well as through immunofluorescence recordings of microtubules in a complex epithelial tissue. Here, we applied a recently proposed deconvolution approach and showed that images obtained from time-gated pulsed STED microscopy may benefit concerning the signal-to-background ratio, from the joint deconvolution of sub-images with different spatial information which were extracted from offline time gating.

## Introduction

The importance of light microscopy in general and fluorescence microscopy in particular as a biophysical imaging tool for understanding life on the cellular and sub-cellular levels is unarguable [1,2]. The high degree of specificity achievable by fluorescent proteins or by tagging proteins with organic fluorophores along with the mostly noninvasive character of this method are often cited as main reasons for the wide distribution of fluorescence microscopy in the biological and biomedical sciences [2,3]. The main drawback of conventional fluorescence microscopy, especially when investigating cellular functions mediated by the interplay of proteins, is the limitation of the spatial resolution to about half a wavelength of the excitation light. This diffraction barrier, however, has been overcome by the invention and development of super-resolution or diffraction-unlimited fluorescence imaging techniques within the last two decades [4–6]. The manner in which the higher precision of nanoscopic information helps in understanding biological processes has been reviewed recently [7–9].

Sharing the general principle of separating adjacent features by forcing the labeling fluorophores within an area of diffraction-limited size to time-sequential emission, two main groups of nanoscopy implementations are commonly distinguished [10,11]. In the stochastic approaches (e.g. PALM, STORM), the fluorophores are kept in a dark non-emission state most of the time. Only a small fraction, on average less than one molecule per diffraction-limited volume, is allowed to be in the bright, fluorescent state, such that the fluorescence of all single molecules detected by a camera at one time can be localized with diffraction-unlimited precision by centroid calculation. As labeling molecules switch stochastically between bright and dark states, it is possible to reconstruct a super-resolution image through the thousand-fold repeated detection and localization of molecule subsets, sparsely distributed over the sample [6,12].

Being the first realized approach of optical far-field nanoscopy, STED is the main representative of the target-coordinated group of diffraction-unlimited techniques [13]. In contrast to stochastic methods, the spatio-temporal fluorescence emission modulation in this group of techniques is realized using special light patterns at well-defined sample coordinates [14]. In the case of STED, stimulated emission by a laser beam that features at least one zero intensity in the focal plane is employed to prevent fluorescence emission from fluorophores excited by the focused excitation light. Therefore, the diffraction-limited spot of the focused excitation beam is exactly superimposed with a doughnut-shaped focal light distribution by a red-shifted depletion beam, bringing the excited fluorophores to their ground state through stimulated emission, before they can emit fluorescence. Application of sufficient STED-laser intensity in the doughnut crest results in the efficient de-excitation of all fluorophores except those located in the center of the depletion beam. This yields an effective fluorescence spot of sub-diffraction size that can be scanned across the sample to achieve diffraction-unlimited imaging [15].

Structured illumination microscopy (SIM) is normally not considered a diffraction-unlimited nanoscopy method, but is still capable of improving the spatial resolution to about one-half of the diffraction limit [16]. Here, several images of one specimen are acquired after excitation of the fluorophores with a series of spatial light patterns in wide-field mode. Linear post-processing of the images enables the recovery of higher spatial frequencies resulting in a reconstruction with twice the normal resolution. Although the resolution enhancement is moderate, SIM is of great importance for imaging living tissues when a long-term time course, a large field of view and low phototoxicity are desirable [1,17].

In principle, the different nanoscopy techniques available nowadays should allow for most of the relevant imaging modalities initially developed for conventional far-field microscopy, but now at higher spatial resolution. The individual techniques, however, appear differently well-suited or technically demanding for the implementation of an imaging modality when applying e.g. two-photon excitation [18], fluorescence lifetime imaging [19], or dynamic single molecule tracking [20]. Non-invasive, sub-diffractional multicolor and/or 3D-imaging of biological samples under physiological conditions has been demonstrated by applying techniques from both the coordinate-targeted method and the individual molecule localization method [21–26].

While the 3D-imaging and multicolor (by spectral separation) modality can be argued to be most easily integrated into a STORM or PALM microscope, STED nanoscopy seems particularly well-suited when it comes to diffraction-unlimited fluorescence lifetime imaging [19] or to spectroscopy at the molecular level [27]. The numerous realized biological applications with impressive results previously unachievable, as well as the great potential for further technical developments are the reasons for awarding the Nobel Prize in Chemistry 2014 to the inventors of diffraction-unlimited fluorescence microscopy [28].

One may argue if today, where different super-resolution microscopes are already commercially available, it is still reasonable to buy a research-grade fluorescence microscope without super-resolution option at all. Actually, STED modality (based on the easySTED principle) has also been added to the MicroTime 200, an old version of which served as a basis for the upgrade in this work. We wondered about a different aspect of this development: Have thousands of high-end microscopes used in research today become obsolete due to the upcoming super-resolution?

Only relatively basic modifications are necessary to use standard wide-field microscopes (not necessarily, but most simply, in total internal reflection fluorescence (TIRF) modality) for diffraction-unlimited imaging applying the single molecule localization method, since it relies more on the photochemical properties of the fluorescent dye used and the subsequent localization analysis algorithms [29]. The corresponding choice to upgrade a confocal scanning microscope would be the STED technology. Here, we explore the possibility of upgrading a commercial time-resolved confocal scanning microscope [30] to perform time-resolved fluorescence imaging of diffraction-unlimited spatial resolution, keeping technical and financial demands at a relatively low level.

Several designs for the realization of STED microscopes have been published in the last few years [31–35]. While, on the one hand, the improvement of spatial resolution, the reduction of photobleaching, and the enhancement of acquisition speed have been in the focus of technological development [36–39], lately, on the other hand, emphasis has also been given to reducing complexity in order to make the technique more popular [40,41]. One development worth noting in this latter context is the commercial availability of an easy-STED phase plate that creates the STED doughnut light distribution in the focal plane while leaving the excitation beam unaltered when implemented into the joint beam path of excitation and STED light [42,43]. This offers the convenient possibility to coalign and spatially filter both beams by coupling them into the same single-mode fiber, thus reducing the alignment of the excitation and STED spot to the joint coupling into this common fiber. We successfully applied such a phase plate to upgrade a time-resolved confocal microscope (MicroTime 200, PicoQuant, Berlin, Germany) by adding just a handful of optical elements and a turn-key STED laser [44] to achieve diffraction-unlimited imaging with spatial resolution down to 40–50 nm.

## Materials and Methods

### STED upgrade of the confocal microscope

**Fig 1:**
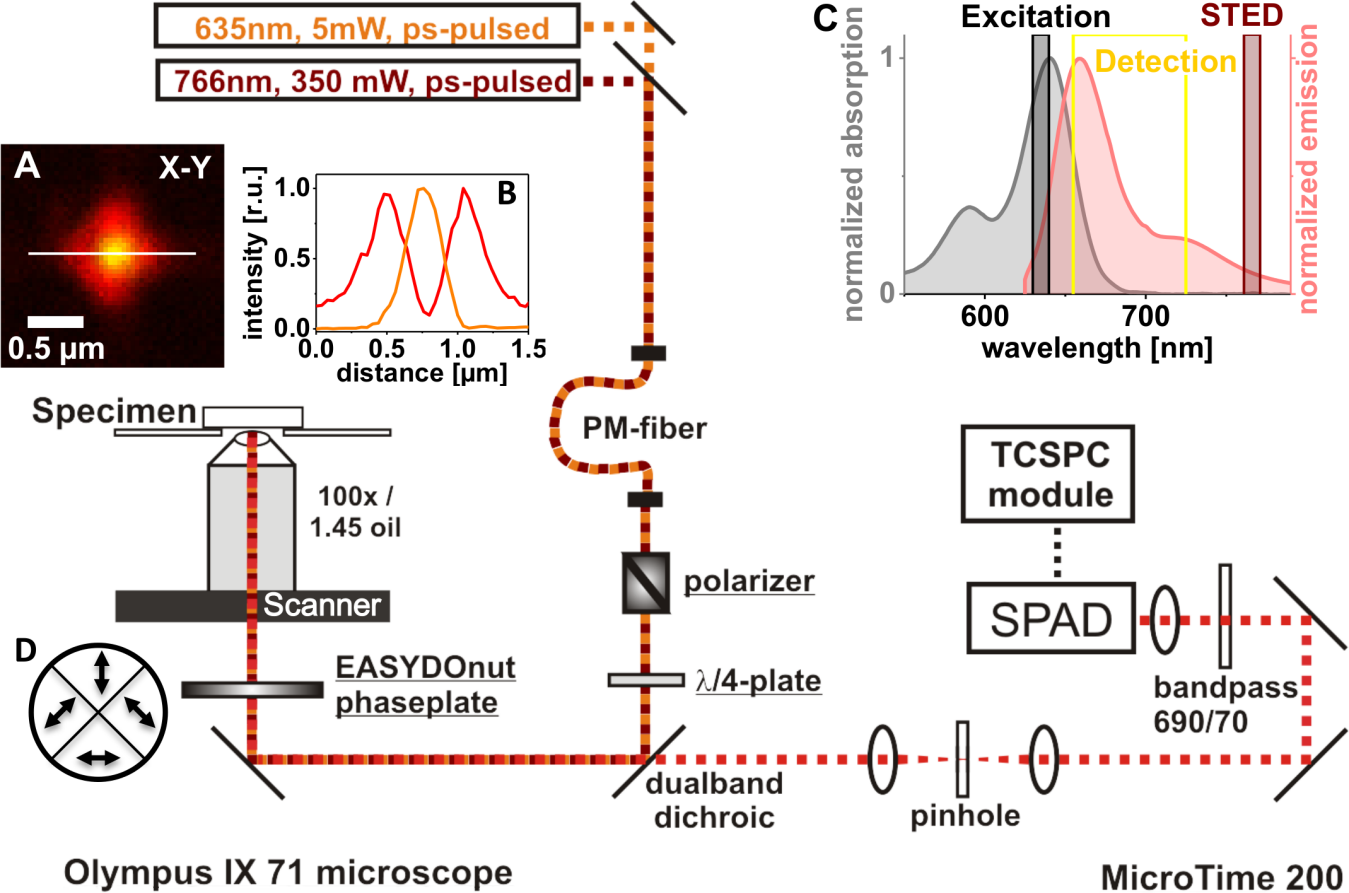
Setup realizing a STED upgrade. Components integrated into the MicroTime 200 confocal microscope (PicoQuant, Germany) in order to reach diffraction-unlimited imaging are underlined. The laser beam inducing stimulated emission (766 nm) is coupled into the same polarization-maintaining (PM) fiber as the excitation light from the ps-pulsed laser diode at 635 nm. The polarizer (Glan-Thompson prism from Artifex, Germany) is not mandatory, as the linear polarization of the lasers used is preserved mostly by the fiber. Still, as the quality of the STED doughnut created by the EASYDOnut phase plate (Abberior, Germany) depends on the polarization state of the 766 nm light, further polarization control may be beneficial. Arrows in inset (D) indicate the slow axis’ direction of each of the phase plate’s segments. The achromatic quarter wave plate (λ/4) changes the polarization state to circular, the state the phase plate works best with. Fluorescence was recorded with a 100 Plan Apo Lambda oil immersion objective from Nikon. Time-correlated single-photon counting (TCSPC) electronics and analysis software were provided by PicoQuant. For single-photon detection, a single-photon avalanche diode (SPAD, PerkinElmer) was used. Inset (A) shows the point spread functions of the excitation (yellow) and STED (red) light as measured by monitoring the backscattered light of an 80 nm gold bead. Inset (B) shows the corresponding profile plots along the white line in (A). In inset (C), the absorption and emission spectra of the dye Abberior STAR635 are given together with the wavelengths of the chosen excitation and STED lasers. The detection window, realized by a 690/70 nm bandpass filter in front of the detector, is indicated as a yellow box.

An inverted time-resolved confocal scanning microscope (MicroTime 200, PicoQuant, Berlin, Germany) served as a basis for the upgrade to a super-resolution microscope (Fig 1) [30]. In order to realize STED-based imaging in a confocal fluorescence microscope, it is, in principle, only necessary to spatially overlay the focal spot of the excitation laser beam with a red-shifted light distribution realizing a diffraction-limited focal zero-intensity minimum in its center. Both continuous-wave [45] and pulsed STED laser sources are convenient for the upgrade, with the pulsed option promising higher achievable resolution at the same average laser intensity [46,47].

Relying on trains of excitation and depletion pulses, the pulsed modality requires thorough temporal synchronization to guarantee the simultaneous or shortly delayed (with respect to the excitation pulse) arrival of the STED pulse in the focal plane. While in the early implementations of STED microscopy often Ti:Sapphire laser systems were used to deliver the pulsed STED light [36,48,49], nowadays more compact laser sources are available in the relevant wavelength regions at much lower costs [33,43]. For an efficient STED process, the typical fs pulses of a mode-locked Ti:Sapphire laser needed to be stretched in the past to avoid problems caused by polarization effects, timing jitter and multiphoton excitation partly responsible for photobleaching of fluorescent dyes used. Today’s commercial availability of (turn-key) fiber lasers that already have STED-compatible pulse-widths of 0.1–1 ns significantly reduces the technical complexity of setting up a (pulsed) STED microscope.

As a STED laser source, we have tested a newly developed fiber-amplified laser diode LDH-P-FA-765 (PicoQuant, Berlin, Germany) that delivers an average power of up to 350 mW at 40 MHz repetition rate at a wavelength of (766 ± 3) nm [44]. The repetition rate can be freely chosen between 1–80 MHz. The pulse width is about 80 ps, which is at the lower limit of the 100–1000 ps regime in which the best STED efficiency is expected [46]. Recently, a modified fiber-amplified and frequency doubled laser diode from PicoQuant and fiber lasers from other manufacturers are available that exhibit pulse widths of several hundred picoseconds. These longer STED pulses (>300 ps) have been reported to be beneficial concerning the challenge of controlling photobleaching of marker dyes during the STED run [43]. Longer pulses may also simplify the temporal synchronization of the experiment, as the exact delay of the STED pulse becomes less critical for the performance of the microscope. Still, the 80 ps-pulses used here are already appropriate for STED [50,51], and we achieved reasonable results, indeed. At any rate, we note that there is potential for further improvement in the future.

For the excitation of marker dyes, a 635 nm laser diode (PicoQuant) with an average power of approx. 5 mW was used. Both laser heads (excitation and depletion) were driven by a multichannel picoseconds laser driver (PDL 828 Sepia II, PicoQuant) and triggered by the same oscillator module with a maximal repetition rate of 40 MHz. Time delay was thus very stable and could be controlled to an accuracy of 50 ps via an adjustable electronic delay. The joint coupling of both laser beams into the very same single-mode polarization-maintaining fiber (630PM; Coastal Connections, CA, USA) enabled the mechanical de-coupling of the excitation sources from the specimen and the detection unit.

The starting point when upgrading an existing microscope is slightly different from designing and constructing a new nanoscope from scratch. One aspect is the limited space for the integration of new optical components. The use of a single-beam path setup, in which excitation and STED beam are aligned by coupling into the same single mode fiber, offers advantages here. At the exit of the fiber, the beams are perfectly overlaid and co-linearly aligned if chromatic effects at the out-coupling collimation optics have a negligible influence on the light leaving the fiber. Thus, there is no need to introduce further optical elements to the (limited) space of the main optical unit of the microscope for focus adjustment or special *xy*-adjustment of the STED beam.

The drawback of this coupling into only one fiber, on the other hand, is the very limited possibility to correct for displacement of the two beams due to chromatic effects induced by the remaining optical elements in the joint optical path. Thus, chromatic effects should be eliminated from the very beginning by using achromatic or apochromatic devices for collimation and polarization control.

An α-BBO Glan-Thompson polarizer with an extinction ratio <510^−6^ (no. 50.230.00010; Artifex Engineering, Emden, Germany) for rejecting STED light of polarization different from the desired linear fraction was introduced into the setup’s beam path. As the fiber is polarization-maintaining, this polarizer is not absolutely necessary as long as the orientation of the fiber’s slow axis is carefully aligned with the polarization of the STED laser. Additionally, the effect of mode mixing with the fast axis due to mechanical stress on the fiber should be minimized by controlling the polarization state at the exit of the fiber if the Glan-Thompson polarizer is removed.

An achromatic quarter wave plate (working range: 500–800 nm; Artifex Engineering, Emden, Germany) was positioned in the light path in order to optimize the obtained circular polarization of the 766 nm STED-light at the position of the segmented phase plate close to the back aperture of the objective. For the separation of excitation and emission light, the dichroic mirror exhibited a second reflection band at the STED wavelength. We chose a dualband dichroic (zt635/766rpc, AHF Analysentechnik, Tübingen, Germany) to realize the reflection of excitation and STED light and transmission of the fluorescence signal in the spectral range from 650–750 nm (and 780–920 nm). All optical elements integrated into the beam path were purchased at λ/10 quality, to minimize distortion of the wave front.

The segmented phase plate that forms the focal STED doughnut from the 766 nm laser beam, while leaving the Gaussian profile of the excitation light at 635 nm practically unaltered, has been described in detail [42,43] and is commercially available (EASYDOnut phase plate 635 nm/765 nm, Abberior, Göttingen, Germany). The four segments of birefringent material, each oriented in a way ensuring the slow axes of opposite segments form a right angle, are combined to form the wave plate. Arrows in Fig 1D indicate the corresponding directions of the slow axes. The thickness is chosen such that 765 nm light polarized along the slow axis is retarded by 2.5 wavelengths, while the retardance for the 635 nm excitation light is 3.0 wavelengths. The polarization of the primarily circular polarized 765 nm light is manipulated in a way resulting in the formation of a doughnut-like intensity distribution in the focal plane [42]. Fig 1A shows in red the backscattered STED light from a gold nanoparticle with a diameter of 80 nm (BBI Solutions, Cardiff, UK) in the focal plane of the objective (Nikon CFI P-Apo 100x Lambda Oil, NA 1.45, WD 0.13). The intensity distribution of the backscattered light at 635 nm is shown in orange. The focal STED light distribution is not a perfect round doughnut, as it can be created with a helical phase ramp [52], but rather shows a fourfold symmetry (which is a consequence of the four segments of the phase plate). Important, however, is the fact that a zero intensity in the center is surrounded by high light intensity in any direction. The EASYDOnut phase plate was mounted close to the *xy*-piezoscanner with an inhouse made holder that allows micrometer-fine positioning of the *xy*-position of the phase plate relative to the laser beams.

The chosen wavelength combination (635 nm excitation and 766 nm stimulated emission) is adequate for STED imaging of different dyes having excitation maxima in the red domain of the visible spectrum. Fig 1C shows as an example the absorption and emission spectra of the dye STAR635 (Abberior, Göttingen, Germany). The wavelength ranges of the lasers used are indicated, as well as the detection window used here for STED microscopy. Another dye that has also been used successfully for STED is Atto647N (Atto-Tec, Siegen, Germany). Showing similar spectral properties, with an absorption maximum of 644 nm close to the excitation wavelength and a broad emission with a maximum at 669 nm, this dye also ensures sufficient STED probability.

The microscope was equipped with an *xyz* piezo-scanner (Physik Instrumente, Karlsruhe, Germany) that moves the objective for 2D-imaging within an area of maximally 80×80 μm^2^. Even though the scan range is small compared to the beam diameter of 4 mm (1/e^2^-optical power diameter) overfilling the objective by less than 1%, objective scanning may raise the question of possible misalignment effects on the shape and relative position of the two PSFs in the focal plane. To address that question, we imaged the backscattered light from 80 nm gold beads at different positions along the extreme periphery of the scan range (S1 Fig). However, no indication of degradation of the central minimum or intensity decrease of the maxima toward the periphery of the scan range could be obtained.

Fluorescence was detected confocally (pinhole diameter of 50 μm) with a single-photon avalanche photodiode (SPAD, SPCM-AQR-13, Perkin Elmer, Waltham, USA). Time-correlated single-photon counting (TCSPC) was performed by the module PicoHarp 300 with time resolution down to 4 ps, and fluorescence decay analysis occurred using the software SymPhoTime 64 ver. 1.6 (both PicoQuant, Berlin, Germany). Modification of imaging software for confocal microscopy (here, PicoQuant’s SymPhoTime 64) was not necessary, as rendering images in both confocal scanning mode and STED mode are identical. Only the pixel size needs to be chosen smaller for diffraction-unlimited imaging, accordingly.

The overall cost for components necessary for the upgrade (see S1 Table for a list of components), added up to almost 50,000 Euro (excluding tax and shipping costs), with the high power STED laser accounting for the biggest part. We note that our MicroTime 200 had already been equipped with the 635 nm excitation laser diode, the corresponding fiber coupler and the TCSPC module for time resolved measurements. Coupling the STED laser into the single mode fiber with two mirrors in precision kinematic mounts reaching high coupling efficiency is perhaps the most time-consuming task in the upgrade. After changing the dichroic mirror to the STED compatible one and controlling the polarization state of the light at the exit of the fiber and at the position of the segmented phase plate, fine adjustment of the light path in the microscope can be done with the 635 nm excitation light only as it is the standard way for confocal microscopy. Coarse lateral alignment of the phase plate can be done using the internal lamp of the microscope in transmitted light mode. The borders between the four segments of the phase plate can be made visible as a cross at a small setting of the field diaphragm by lowering the condenser towards the phase plate. Super-imposing the center of the segmented phase plate and the crosshair in the eyepiece will yield a well-aligned phase plate given the microscope is also well aligned. Fine adjustment of the phase plate was done by imaging the backscattered light of gold beads (S1 Fig).

### Sample preparation

Bead probes for the determination of the spatial resolution were prepared by immobilizing 20 nm Crimson fluorescent beads (Life Technologies, Darmstadt, Germany) on (170±5) μm thick high-precision coverslips treated with 0.1% poly-L-lysine (Plano, Wetzlar, Germany) and subsequent embedding in Mowiol 4–88 (Carl Roth, Karlsruhe, Germany).

*In vitro* samples of F-actin filament networks were prepared by immobilization of pre-formed F-actin filaments purified from rabbit skeletal muscle (Cytoskeleton, Denver, USA) on a coverslip treated with 0.1% poly-L-lysine. Before labeling with phalloidin-coupled fluorescent dyes (either STAR635 or Atto647N), a blocking step using 2% bovine serum albumin (BSA, Sigma-Aldrich, Deisenhofen, Germany) was performed to reduce non-specific dye binding. After 60 min of dye incubation in a final concentration of about 200 nM, residual dye molecules were eliminated by three washing steps in phosphate-buffered saline (PBS), and the specimen was subsequently embedded in Mowiol 4–88 containing the anti-bleaching reagent DABCO (25 mg/ml; Carl Roth, Karlsruhe, Germany).

Cryosections from salivary glands (10 μm thick) of blowflies, which were reared at the Dept. of Animal Physiology (Univ. Potsdam), were prepared as described previously [53]. For direct staining of F-actin cryosections were incubated for 1 h with STAR635-phalloidin (1:40). For indirect immunofluorescence staining of microtubules, the cryosections were incubated overnight at 4 C with the rat α-tubulin antibody YL1/2 (1:100; Thermo Fisher Scientific, Schwerte, Germany) and subsequently reacted for 1 h with the goat anti-rat IgG secondary antibody (1:200; Life Technologies, Darmstadt, Germany) conjugated to the fluorescent dye STAR635P (Abberior, Göttingen, Germany). To avoid mechanical stress to the cryosections, spacers were used when embedding the specimen in Mowiol 4–88 containing 25 mg/ml DABCO.

### Time-gating and joint deconvolution approach

By performing rasterscanned imaging in combination with TCSPC detection, the whole temporal information of the fluorescence emission of the excited fluorophores was recorded for every image pixel (chosen time resolution of 8 ps resulted in a total time window of ~33 ns.) This allowed for off-line time-gating of the pulsed STED images by simply selecting those photons recorded in a time-gate (of variable length) shifted by a (variable) time interval *Δt* relative to the excitation pulse [32,38]. In our implementation of STED (using excitation and STED pulses of approx. 100 ps length), time-gating could considerably improve the quality of diffraction-unlimited images concerning the signal-to-background ratio (SBR) and thus indirectly the resulting resolution, if this was background-limited. By setting a detection time gate starting at the end of the STED pulse, for example, the SBR could be significantly increased, as background contributions due to scattering of the excitation and STED pulses were discarded.

In a perfect time sequence for a pulsed STED experiment, (rectangular-shaped) pulses that are very short compared to the fluorescence lifetime of the fluorophore should interact with the specimen in a sequence of the STED pulse following the excitation pulse with neither a temporal delay nor an overlap. In such an idealized case, fluorophores are excited and de-excited on a timescale that is short enough to keep the level of spontaneous fluorescence from the periphery of the doughnut-formed point spread function (PSF) during the interaction of the pulses negligibly low. An increasing pulse length as well as any delay of the STED pulse with respect to the excitation pulse will increase the level of unwanted spontaneous emission from the diffraction-limited excitation PSF-volume. Any temporal overlap of the two pulses, on the other hand, will diminish the efficiency of the overall STED process, as the suppression of fluorescence depends on the number of STED photons to which the molecule is exposed while residing in the excited state [47]. However, excessively short STED pulses (<100 ps) have been observed to increase the technical complexity concerning the temporal synchronization. Synchronization jitter may be problematic when using laser diodes [46]. More importantly, short pulses with their higher peak powers are more demanding to the fluorophore with respect to photobleaching. As it is one known channel for permanent photobleaching, the rising probability of multiphoton excitation at higher peak powers may be cited to explain this effect of enhanced photodamage at shorter STED pulse lengths [50,54,55].

In a practical application, using approx. 100-ps pulses and avoiding a major temporal overlap of excitation and STED pulse (thus using the available STED power most efficiently for stimulated fluorescence quenching), STED images could always be improved by time gating and discarding early photons emitted during the application of the laser pulses. The time-synchronization protocol of laser flashes and the length and temporal shape of the laser pulses determine how the spatial information gained from photons collected in different time gates varies. The first photons detected after laser excitation (and before the application of the STED pulse) result in diffraction-limited images with spatial information identical to that of a confocal imaging modality. Depending on the instrumental response of the detection system and the temporal shape of the STED pulse, the photons with the highest diffraction-unlimited spatial information are collected with a certain delay to the end of the STED interaction.

The emission detected in the remaining intermediate time gate reveals a confinement to the center (of the depletion doughnut) that rises as a function of time delay relative to the laser pulse. Similarly to the case of continuous wave (cw)-STED, this effect can be described by a time-dependent effective PSF of the STED system (in this time gate), that may be modeled as the weighted sum of different Gaussian distributions with decreasing full width at half maximum (FWHM) and decreasing weight [47]. Accordingly, the image resulting from those photons can be imagined to be the sum of a stack of sub-images with different spatial resolutions, starting at diffraction-limited resolution and converging toward the maximally achievable spatial resolution of the highest resolved STED time gate.

All time gates described contain the spatial information of more or less resolved images of the specimen. The best resolved gated STED image can be obtained simply by disregarding all the photons arriving outside the “last” time gate. This procedure is unsatisfactory, however, as the information from all the detected emission outside the gate is lost. It has thus been proposed that diffraction-unlimited gated cw-STED images be further improved by methods of multi-image deconvolution that take into account the time-dependent effective PSF of cw-STED microscopy [47,56].

**Fig 2:**
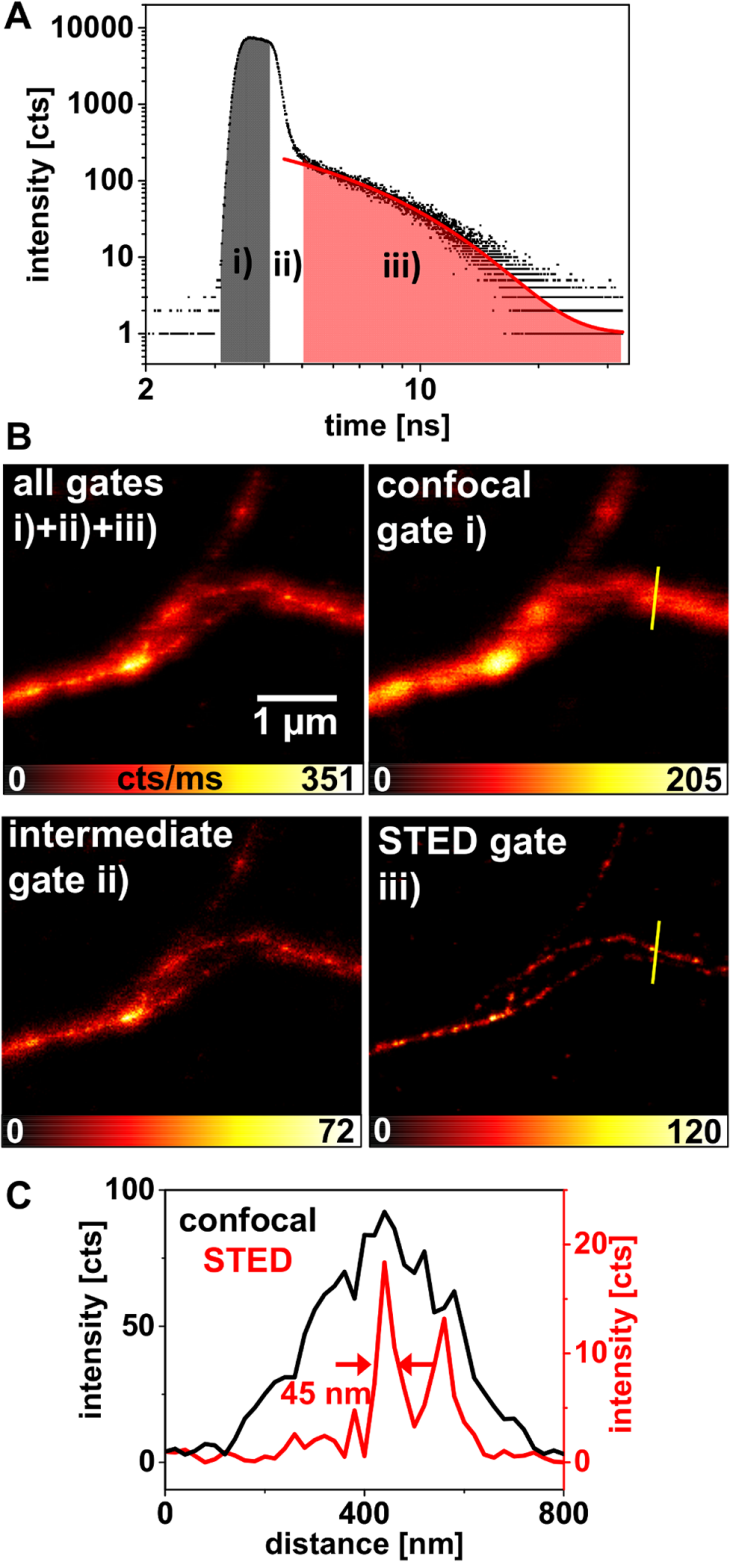
TCSPC approach for time-gated STED imaging. (A) Double-logarithmic TCSPC histogram of the fluorescence signal of the F-actin marker Abberior STAR635-phalloidin under ps-pulsed excitation and ps-pulsed stimulated emission. Three time gates are indicated: gate *i)* contains the fluorescence emitted by the labeling dye before application of the STED pulse, the gate *ii)* intermediate gate contains residual fluorescence emitted during the stimulated emission process, and the gate *iii)* STED gate contains the fluorescence from the very center of the STED doughnut after the completion of the stimulated emission process. (B) Fluorescence images of STAR635-phalloidin labeled *in vitro* F-actin obtained from the different time gates *i)*, *ii)*, *iii)*, and the sum of all three time gates, as indicated in (A). Gate *i)* comprises photons with the confocal spatial information of the structure, gate *iii)* photons with the diffraction-unlimited STED information, and gate *ii)* contains a mixture of fluorescence photons that differ in the spatial information they carry. (C) Profile plots along the yellow line in the confocal and the STED image in (B), indicating the same F-actin structure with different spatial resolutions.

The ‘time-dependent’ spatial information of the same structure, independently accessible in different time gates of the pulsed STED modality (as described here), calls for the application of similar strategies to further optimize the gain of information. While the effective PSFs of the images constructed from the early emission after excitation (gate *i)* in Fig 2A) and the late emission after completed de-excitation (gate *iii)* in Fig 2A) are mostly time independent and can therefore be determined rather easily; this is not the case for the intermediate photons (gate *ii)* in Fig 2A). We therefore decided to test an adaption of a multi-image deconvolution whose usage for super-resolution microscopy has recently been suggested by Ingaramo and coworkers [57]. Based on the Richardson-Lucy (RL) algorithm [58,59], this approach enables joint deconvolution of images of the same structure at different resolutions and different brightness levels. We jointly processed the two images comprising time gate *i)* with confocal resolution and gate *iii)* with diffraction-unlimited STED resolution through the slightly modified implementation of this approach in the python programming language provided by Ingaramo and coworkers. Photons collected in gate *ii)* were discarded from image post-processing in this study due to the more complex and time-dependent PSF of the system in this time gate, as well as to the fact that scattering light from the STED laser corrupts the image exclusively in this gate.

While the confocal PSF determining the blurring of the early photons can be described well by a Gaussian [60], this is often not the case for the effective PSF of the STED imaging modality. Due to a non-perfect depletion of emission from the doughnut periphery, the PSF of the STED system, as visualized by imaging nanobeads, shows strong pedestals [51]. Incomplete depletion due to early photons (vide supra) can effectively be countered by time gating, while residual fluorescence, also excited by the STED pulse itself, may explain the pedestals of the PSF of time-gated STED images. In STED images of this study, the light distributions of structures with a diameter smaller than the actual resolution of the STED system were thus more adequately simulated by Lorentzian peak functions than by Gaussian-shaped peaks (S2 Fig).

The simultaneous reconstruction of multiple images (blurred and corrupted by noise to different degrees) of the same object through multi-image deconvolution has shown its potential to improve signal analysis in microscopy [56,57,61]. For an imaging process of the form *m* = *P*(*h* ○ *o*) with *m* being the measured image, *o* the imaged structure (distribution of fluorophores), *h* the system PSF (modeling the imaging process by convolution ○), and *P* a representation of the Poisson noise distribution, the Richardson-Lucy deconvolution iteratively reconstructs the most probable density estimate, *e*, for *o*, from the measurement *m*, and the known PSF, *h*. Being a relatively simple realization of the maximum-likelihood approach adapted to Poisson noise [62], one iteration of a two-image RL deconvolution, as applied here, may be written in the form of a matrix equation according to [56]:

$$e^{l+1}=e^{l}\;\left(\frac{1}{2}\sum_{i=1}^{2}\;H_{i}^{T}\frac{m_{i}}{H_{i}\;e^{l}}\right)$$ (1)

In the case of 2D-image processing, the estimates at iteration *l*, *e* ^*l*^, were represented by *u*×*v* matrices of the same size as the measured input image, *m*. *H*_*i*_ stands for the operator performing the discrete convolution with PSF *h*_*i*_; *H* ^*T*^ is the corresponding convolution with the flipped PSF *h* ^T^ *(u*,*v)* = *h(-u*,*-v)*. Multiplication and division of matrices in Eq (1) are computed element-wise.

The adaption of the joint deconvolution approach used in this study comprised basically two changes to the algorithm proposed by Ingaramo et al. First, instead of using two Gaussian functions differing in the FWHM as PSFs for the deconvolution of the two images, we used one Gaussian with diffraction-limited FWHM and one 2D-Lorentzian peak function of width *w* as PSF for the STED image (S2 Fig):

$$h_{2}\left(x,y\right)=\frac{1}{\left(1+{\left(\frac{x}{w}\right)}^{2}\right)}\frac{1}{\left(1+{\left(\frac{y}{w}\right)}^{2}\right)}\frac{1}{\left(w^{2}\pi^{2}\right)}.$$ (2)

Second, a weighting factor was tentatively introduced to adjust the relative influence of diffraction-limited and diffraction-unlimited image to the reconstructed image, as has been proposed similarly by Verveer and Jovin [61]. By scaling the input images in Eq (1) according to *ḿ*_i_ = *c*_i_ *m*_i_, a ratio *c*_1_/*c*_2_ > 1 will result in an increased relative importance of the confocal sub-image. In some circumstances (one example where the STED signal of a filamentous structure is very low is discussed in S3 Fig), the SBR of selected features in the convolved image were found to benefit from an increased importance of the confocal sub-image. However, the addition of a further parameter to the algorithm that is promoted for having only one parameter (the number of iterations) to be adjusted [57] counters the ease of use of the joint deconvolution approach. As we have not further investigated the nontrivial problem of estimating an optimal value for this scaling parameter (caption in S3 Fig), we confined ourselves to showing that the SBR of a biological specimen may be increased by the joint deconvolution in the form given in Eq (1) (without relative scaling of the input images) compared to a conventional deconvolution (S4 Fig).

The widths *w*_*i*_ of the Gaussian- and Lorentzian-shaped PSFs entering the deconvolution algorithm as a parameter are ideally determined from the input sub-images to be processed themselves, as the diameter of the STED PSF depends on the depletion laser power and the spectral emission of the applied dye at the STED wavelength. In the case of imaging F-actin (filament diameter of ~7 nm) stained with small dye-phalloidin conjugates, it can be assumed that the diameter of the fluorophore distribution perpendicular to the filament is clearly below 20 nm [63], thus allowing the determination of the PSF diameter (>50 nm) from cross-sections of the fluorescence image of a single filament (S5 Fig).

Microtubules having an outer diameter of ~25 nm and stained via indirect immunofluorescence are not adequate for the determination of a STED PSF of the width of ~50 nm, as the fluorescence-emitting structure itself has a similar diameter. To deconvolve images of microtubules, we therefore determined the PSF from F-actin images measured at the same STED intensity as the tubule images (S4 and S5 Figs).

## Results and Discussion

### Spatial resolution

**Fig 3:**
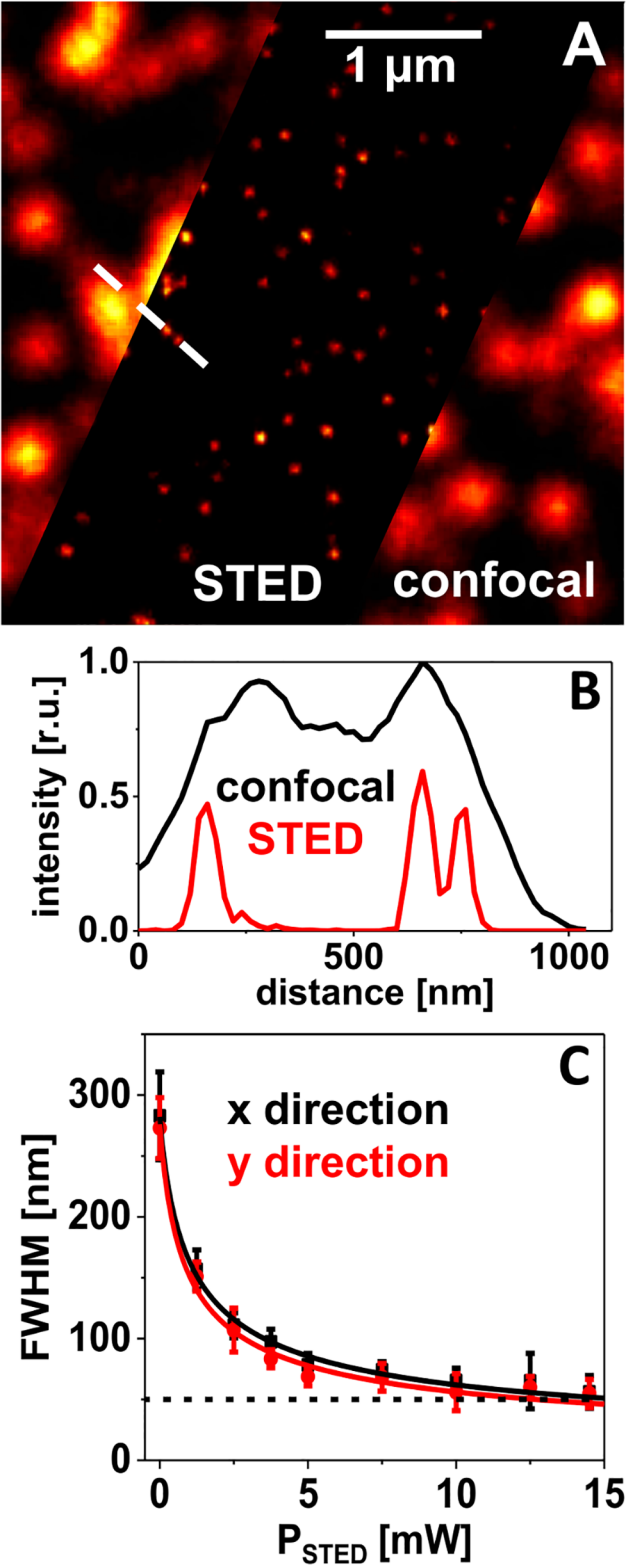
Diffraction-unlimited imaging of crimson beads. **(**A) Confocal and STED image of the same preparation of 20 nm crimson beads on a glass substrate, embedded in Mowiol/DABCO. STED power 14.5 mW (at the objective’s back aperture) at a repetition rate of 2.5 MHz. (B) Profile plots along the dashed white line in (A). (C) Full width at half maximum (FWHM) values determined by fitting 2D-Gaussian single peaks to STED images taken at different STED powers. Each data point represents the mean FWHM value of 10–20 peaks with the corresponding standard deviation. The solid lines show the result of a simulation with a function proportional to one over the square root of the STED intensity (Eq 3).

To estimate the achievable spatial resolution of a microscopic system, beads are often used whose diameter is smaller than the anticipated resolution. We used a sample of crimson beads with a diameter of 20 nm. In Fig 3, images of the same sample with crimson beads on a glass coverslip, measured in both conventional confocal mode and gated STED mode, are compared. In the STED image, only fluorescence is considered that was detected after the STED process was completed (see gate *iii)* in Fig 2A). The two laser diodes were driven at a repetition rate of 2.5 MHz for these measurements. By lowering the frequency of excitation and STED de-excitation from the maximal possible value of 40 MHz to 2.5 MHz the pulse energy delivered by our STED laser rose by a factor of approx. 2. The maximal average optical power of the 766 nm laser at 40 MHz was about 350 mW. Due to coupling losses at the entrance of the single-mode fiber and the summation of reflections at the filters, (dichroic) mirrors, and the objective in the optical path, only a power of about 130 mW was detected at the back aperture of the imaging objective. By lowering the repetition rate and thereby enlarging the peak power of each STED pulse and thus enlarging the STED efficiency, the spatial resolution could be further improved (see S6 Fig for a direct comparison of STED images acquired at laser repetition rates of 2.5 MHz or 40 MHz and otherwise identical experimental settings).

The spatial resolution in images of bead samples is generally determined as the FWHM of intensity peaks of single beads. The exemplarily chosen profile plot (Fig 3B) demonstrates the gain in information from the STED image as features become visible that cannot be resolved in the corresponding confocal image. The resolution of a STED microscope is theoretically expected to scale as the inverse square root of the applied STED intensity, *I*_STED_ [15] according to Eq (3).

$$FWHM\propto \frac{\lambda }{NA}{\left(1+\frac{I_{STED}}{I_{S}}\right)}^{-\frac{1}{2}}$$ (3)

The parameters thus determining the final spatial resolution are the wavelength λ, the objective’s numerical aperture NA and the fluorophore specific saturation intensity *I*_S_, which is often chosen to be the STED intensity guaranteeing 50% de-excitation probability.

From Fig 3C it becomes obvious that this relation is fulfilled quite well for this sample of crimson beads. Here, the mean FWHM values, determined by fitting 2D-Gaussians to an ensemble of single peaks in STED images taken at different STED powers, are plotted. The solid lines represent the result of simulations (least square fits) with the model described by Eq (3). With the STED power available in the present setup (~15 mW at the objective’s back aperture and at a 2.5 MHz laser repetition rate), the crimson beads could be imaged with a resolution of (55 ± 15) nm (N = 12). This was an improvement by a factor of ~5 compared to a FWHM of (280 ± 30) nm in the confocal image.

**Fig 4:**
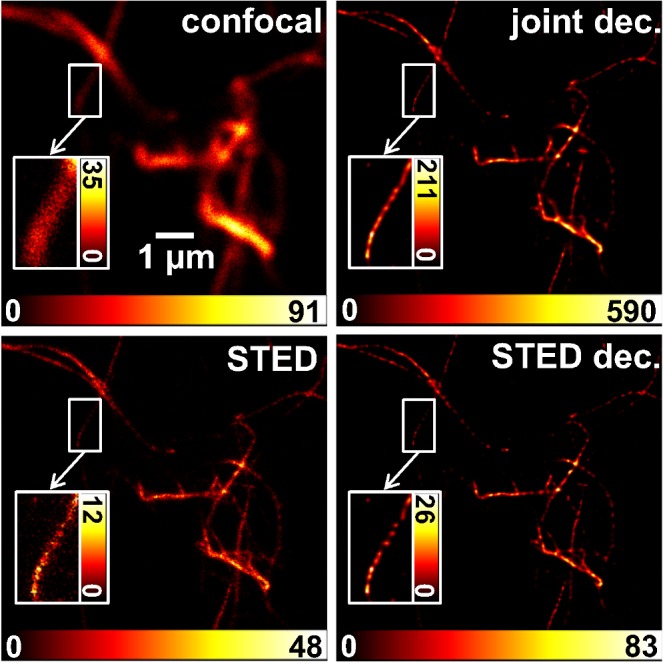
Joint deconvolution of STED images. Left: confocal and STED contribution, derived from offline time gating of the emission from one pulsed STED image acquisition run (STED power of 5 mW at a 2.5 MHz repetition rate) of STAR635-phalloidin labeled *in vitro* F-actin. Right: result of a joint deconvolution of both contributions (joint dec.) and a simple deconvolution of only the STED image (STED dec.); both deconvolutions applied the Richardson-Lucy algorithm. For the joint approach, one PSF of Lorentzian shape with a FWHM of 70 nm and one Gaussian with a FWHM of 300 nm were applied. 108 iterations were performed, and the weighting was 4:1 in favor of the confocal image. For the simple STED-only deconvolution, the same Lorentzian PSF was used as in the joint case, but only 11 iterations were calculated. Insets show the enlarged ROIs at an adapted dynamic range to emphasize tiny differences in the results of the two deconvolution approaches.

**Fig 5:**
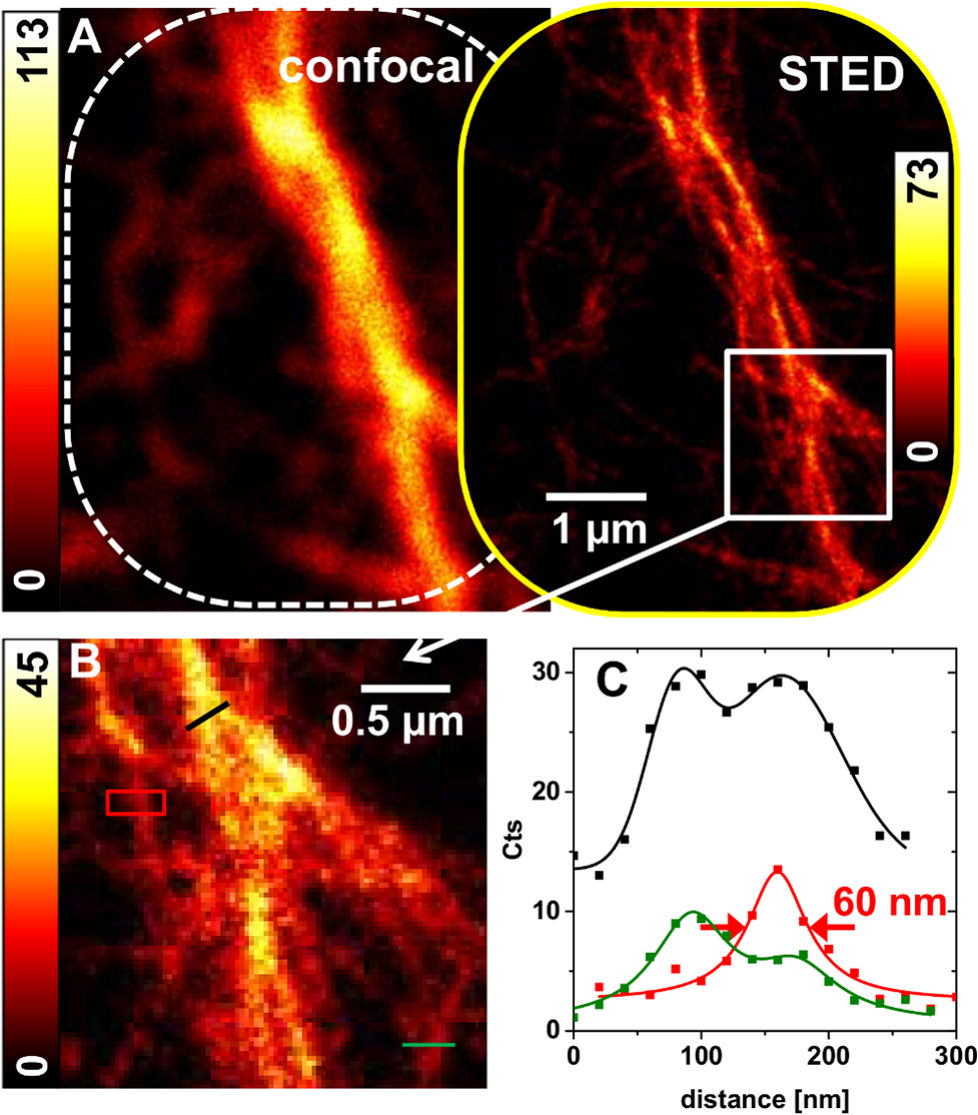
Measurements of *in vitro* F-actin filaments stained with Atto647N-phalloidin. (A) Diffraction-limited and-unlimited images of a gated STED acquisition of a filament structure attached to the glass cover slip and labeled with the dye Atto647N-phalloidin. STED power of 5 mW at a 2.5 MHz repetition rate. (B) The marked region of the STED image in (A) is shown enlarged, and regions of interest (ROI) are marked by lines (black and green) and a red box. (C) Profile plots of the ROIs simulated by single peak (Lorentzian) or double peak functions. The specified FWHM indicates that a single filament is localized to an accuracy of 60 nm. Crossing filamentous structures at a distance of ~80 nm appear distinguishable.

The achieved resolution of approx. 50 nm was relatively robust with regard to fine adjustment of the system. Thus, time-consuming exact alignment in daily maintenance concerning the STED components was not necessary. Particularly an exact time delay between the laser pulses was uncritical if time gating was applied in data evaluation. A lateral displacement of excitation and STED beam in the focal plane of up to 50 nm and a certain deviation from a perfectly symmetric STED light distribution (Fig 1A) were also tolerable. The phalloidin coupled dyes Atto647N-phalloidin and STAR635-phalloidin seemed similarly well suited for STED imaging with our upgraded microscope. STED images of F-actin filaments stained with one or the other dye acquired at a reduced STED intensity of 5 mW (at a repetition rate of 2.5 MHz) are shown in Figs 4 and 5. Profile plots at indicated positions in the STED images (comprising late photons only) indicate that single filaments are localized with FWHM values of 60–70 nm (Fig 5C and S5 Fig).

### TCSPC approach for time-gated STED imaging

The TCSPC histogram of a STED image of F-actin labeled with Abberior STAR635 under ps-pulsed excitation and ps-pulsed stimulated emission is shown in Fig 2A. The timing of the two lasers is directly accessible from this histogram. The rising edge is formed as the convolution of the spontaneous fluorescence emerging from the arriving excitation pulse with the instrument response function (IRF). After having reached its maximum, the fluorescence signal would decay exponentially with the dye typical fluorescence decay time (τ~3.4 ns for STAR635). A much faster decay becomes visible as the stimulated emission is induced by the arriving 766 nm depletion laser pulse. For the measurement shown, the delay of excitation and de-excitation was chosen to be *Δt*~950 ps, as can be seen roughly from the delay of the rising edge to the fast decay, marked as gray region *i)* in Fig 2A. The fast signal decay as a response to the STED process is superimposed over the spontaneous fluorescence signal of those excited molecules in the center of the doughnut that have not been brought to their ground state by stimulated emission. The red line in Fig 2A represents a monoexponential simulation (tail fit) of the fluorescence intensity decay as a function of time in the time gate when the STED process is terminated, marked as red region *iii)*.

Three time gates of overall interest have been indicated in Fig 2A. Gate *i)* contained the spontaneous fluorescence emitted by the labeling dye before application of the STED pulse. The intermediate gate *ii)* contained fluorescence with mixed spatial information emitted during interaction of the STED pulse with the excited dye molecules. Finally, gate *iii)* contained the fluorescence from the very center of the STED doughnut after the completion of the stimulated emission process. In Fig 2B, images of one *in vitro* F-actin structure are shown, for which the photons of the different time gates were taken into account. The best spatially resolved STED image obviously resulted from the fluorescence captured in gate *iii)*. Gate *i)* contained the confocal spatial information of the specimen, as the photons were collected before application of the STED pulse. This TCSPC approach thus has the convenient side effect of delivering a confocal reference image in addition to the STED image. This may be helpful in evaluating the content of a STED image if very low signal intensities are encountered (S3 Fig). Furthermore, lateral misalignment of the focal excitation spot and the focal intensity minimum of the STED PSF can be diagnosed and corrected by taking a look at the offset between STED and confocal reference image. The intensity peak in the STED image of a single bead, for example, should be exactly in the center of the diffraction-limited intensity peak in the confocal image. Lateral misalignment would result in displacement of the STED peak in the corresponding direction. A laterally badly positioned segmented phase plate may cause this misalignment of the focal light intensities and may be corrected by the described offset of STED and confocal image.

A higher brightness of the reference image comes at the cost of a lower brightness of the STED image, though, and is determined by the time delay of excitation and STED pulse, as fluorescent molecules that have already contributed to the confocal image (early time gate) cannot contribute to the STED image in the same excitation depletion turn. Longer delays result in darker STED images. Towards longer time intervals, the achievable diffraction-unlimited resolution is, in theory, not directly dependent on the time delay if time-gated detection is applied [47,51,60]. However, as the STED signal decreases, the signal-to-noise ratio (SNR) becomes more and more resolution limiting.

For the specimen discussed thus far, we were not able to determine a difference in the spatial resolving power when varying the delay between 500–1000 ps. The thinnest actin filaments were imaged with FWHM values between 40–50 nm (Fig 2C). Through application of time-gated detection, constraints on the experiment’s temporal alignment therefore appeared relaxed. A minimum delay time must be kept though, as the efficiency of the STED process suffers from temporal overlap of excitation and de-excitation processes. The shortest possible delay (reaching maximal resolution at maximal brightness in the STED image) thus depends on the length of the laser pulses applied. If excitation and STED pulses are chosen to (partly) overlap in time, the optimal delay should also depend on the fluorophore lifetime and the temporal shape of the pulses.

### Joint deconvolution approach

The joint deconvolution approach, which was applied to the confocal and diffraction-unlimited contributions resulting from different gates of one image acquisition run, relies on parallel performing Richardson-Lucy (RL) iterations [58,59] for both images with two different PSFs, and at the end of each iteration calculating the mean of the two correction terms according to Eq (1) as the basis for the next iteration [57]. A parameter controlling the weight of confocal and STED contribution (by relative scaling of the intensities of the two input images) may be introduced to vary the influences of either part in the minimization process.

The RL algorithm is known to converge towards an amplification of noise, and therefore it is normally stopped after a certain number of iterations [64]. It is not easy to determine the optimal number of iterations; different criteria have been proposed [65], but often it is the subjective decision of the user. The appearance of characteristic spottiness in the reconstructed image with a rising number of performed iterations is usually seen as a convergence of the algorithm towards an implausible solution (dominated by noise) to the ill-posed problem of deconvolution [64,65].

A conventional deconvolution by the RL algorithm of a STED image of F-actin showed such speckle for filaments with low signal intensity (low SNR) after just a few steps (iteration 10 in S7 Fig). Brighter appearing filaments tended to be more robust against the formation of spottiness. Probably due to the higher SNR, they showed a ‘continuous’ signal up to some tens of iterations (S7 Fig). In contrast, for the confocal image at the same pixel size, the algorithm could be iterated several hundred times before amplification of noise became relevant (S8 Fig). Spatial frequencies present in the confocal image are obviously limited to a smaller bandwidth, as visualized by the Fourier transform power spectra shown as insets in S7–S9 Figs. For the joint deconvolution approach, the number of iterations that may be performed without reaching a solution showing the characteristic spottiness (S9 Fig) lay in between these extremes of pure STED or confocal image deconvolution. The joint deconvolution approach was tested firstly by applying it to the images gained by gating the collected emission of pulsed STED images from *in vitro* F-actin filaments. The differences between the classical RL-deconvolution of the STED image only and the joint deconvolution of both differently well resolved images was rather small (Fig 4). Taking a closer look, the insets revealed the reduction of spottiness in the jointly deconvolved image, though. This ‘smoothing’ effect to the reconstructed image was enlarged by increasing the relative scaling of the confocal input image (S3 Fig).

A common way to counter noise amplification in iterative deconvolution is to include constraints based on a-priori knowledge about the structure to exclude implausible spotty solutions to the ill-posed problem of image restoration by deconvolution [62]. Castello et al. have recently evaluated the application of multi-image deconvolution to two sub-images of gated cw-STED microscopy [56]. The second sub-image (with less spatial information) was interpreted as such a constraint on the solution of the RL algorithm. From this point of view, the scaling of the confocal sub-image can be regarded as a regularization parameter that balances between the solution to which the RL algorithm converges from the STED image, and a ‘smoothness’ term defined by the confocal sub-image. Accordingly, the spottiness of the reconstructed image of the single filament is reduced, as the relative scaling factor of the confocal sub-image is increased (S3 Fig).

To have a comparable stopping criterion for different RL deconvolutions, we monitored the width of a characteristic feature in the resulting image as a function of the number of iterations. Here, we chose the intensity distribution along the yellow line in (S3A Fig) that apparently stems from a single actin filament (with a fluorescence active diameter < 20 nm). Corresponding profile plots of chosen iteration steps are shown in the right column of S3 Fig. Specified FWHM values were determined via simulation of the data (symbols) with Gaussian functions (lines). The algorithm was stopped when the FWHM width reached a value of 50 nm that was 2.5 times the pixel size of 20 nm used in the imaging process, which implied a slight improvement over the nominal resolution of 70 nm of the raw STED image (S5 Fig).

The number of iterations that were necessary to reach the anticipated width of 50 nm in the deconvolved image of a single filament rose with increased importance of the confocal image. While only 11 steps were necessary for the STED image alone (S3A Fig), the inclusion of the confocal image at a weighting of 1:1 required 48 iterations to reach the stopping criterion. Weighting the confocal image by 1:2 or 1:4 rose the number of iterations to 74 (S3C Fig) and 108 (S3D Fig), respectively.

The joint multi-image approach thus helped in finding more plausible solutions to the deconvolution problem that benefit from increased SBR compared to the raw image or the conventionally deconvolved image. This effect may be enlarged in some circumstances through relative scaling of the input images. For multi-image deconvolution, it has been proposed to weight different input images according to their SNR [66], but this way the different spatial bandwidths of the images are not being taken into account. Further investigation may reveal an estimate for the optimal choice of the scaling factor as a regularization parameter for time gated pulsed STED images.

**Fig 6:**
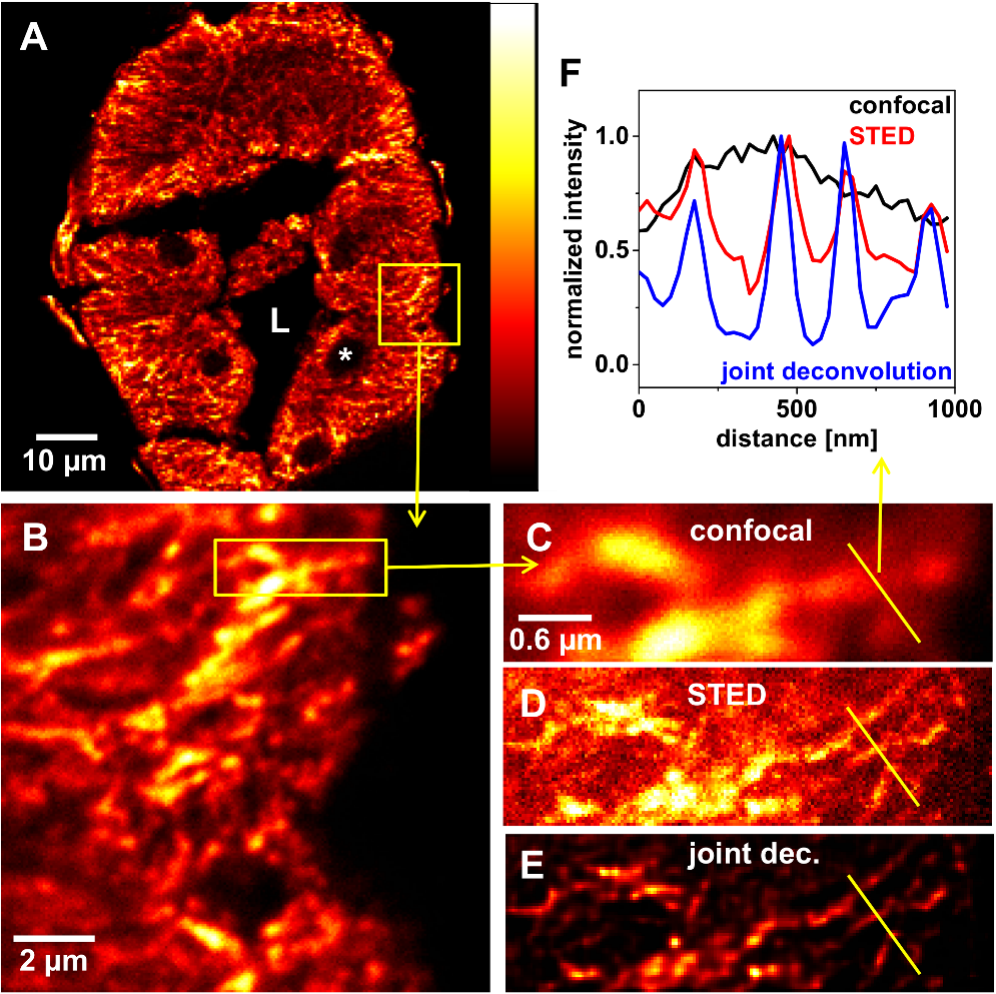
Immunofluorescence localization of microtubules in blowfly salivary glands. Gland cryosections (10 μm thick) were treated with antibody YL1/2 against α-tubulin and fluorescence of secondary antibody coupled to STAR635P was detected. (A-C) A confocal overview image as well as enlarged views of regions indicated as yellow rectangles are shown. The gland lumen (L) and the nuclei (asterisk) are indicated. (D) STED image of the region shown in (C) acquired at a STED power of 5 mW (2.5 MHz). (E) Image with enhanced SBR as a result of a joint deconvolution of both images (C) and (D). Deconvolution parameter: 131 iterations, no scaling of input-images, FWHM values of 70 nm (Lorentzian) and 300 nm (Gaussian). (F) Profile Plots along the yellow lines in (C), (D) and (E), indicating the same microtubule structure with a different spatial resolution.

The *in vitro* preparations of F-actin showed almost no background fluorescence at all (Figs 2 and 4). The general strength of image deconvolution may be seen in the enhancement of the SBR by means of spatial confinement of the signal to its most probable location of origin. STED images of more complex tissue showed much more background fluorescence, probably mostly due to out of focus light. In such images (e.g. Fig 6), the joint deconvolution approach worked very efficiently in improving the SBR (Fig 6E) and was robust against artefacts arising from background fluorescence. A comparison of the reconstructed images of a conventional single image deconvolution of the STED image and of a joint two-images deconvolution is presented in S4 Fig. It can be deduced that the SBR of the imagined microtubule network on top of a rather featureless background is further improved for the joint approach. However, through the enhancement of the SBR, also small features in the image may become invisible as is shown exemplarily in S10 Fig (lower row) for a rather dark area of a gated STED image of F-actin stained with Atto647N-phalloidin. Thus, each deconvolution result should be carefully compared to the raw image and it should be kept in mind that, beside background and noise also badly defined features at low brightness may be suppressed.

### Proof of principle in a complex biological tissue

After demonstration of the proof of principle using well-defined, sparse structures, the realization of diffraction-unlimited imaging from rather complex biosystems is still a demanding challenge. Here, the cytoskeleton of blowfly salivary glands was investigated. Insect salivary glands in general are well-established model systems for studying transepithelial ion transport processes and their underlying intracellular signaling pathways [67,68]. Within the abdominal part of the tubular blowfly salivary glands, a monolayer of secretory cells surround an irregularly-shaped lumen. The apical membrane of these secretory cells is deeply infolded, forming a branched system of so-called canaliculi, which often range up to the basal region of the cell. These canaliculi are further infolded, forming densely packed sheet-like microvilli (so-called microplicae) containing F-actin filament bundles [69]. However, such regularly structured microplicae as previously shown in TEM studies could not be resolved in STED images by using F-actin staining, most probably due to the high packing density of the microplicea (S11 Fig).

In comparison to the above mentioned densely packed bundles of F-actin filaments, microtubules are thought to exhibit a more or less loosened network within the secretory cells. Thus, microtubules have been localized by immunofluorescence confocal microscopy. Fig 6A shows a confocal image of a salivary gland cross-section treated with the primary antibody YL1/2 against α-tubulin. Immunofluorescence could be detected using a secondary antibody coupled to the fluorophore STAR635P. Within the cytosol, a microtubule network could be observed, probably oriented in a basal-apical direction. The nuclei of the secretory cells (asterisk) and the enclosed gland lumen (L) remained unlabeled, as they are free of microtubules (Fig 6A). Even though the microtubule network is sparse compared to the bundles of F-actin in the apical membrane, its three-dimensional extension in combination with the lack of diffraction-unlimited resolution in the *z*-direction made diffraction-unlimited imaging challenging. Still the STED image (Fig 6D) allowed for higher spatial resolution compared to the confocal modality and thus enabled the separation of adjacent microtubules that are closer than the diffraction limit. Strong background light could be further reduced through the joint deconvolution approach (Fig 6E and 6F).

By using thinner cryo-sections one could perhaps minimize the difficulty of degradation of the STED light distribution (central intensity minimum) by scattering and aberration in the inhomogeneous sample that are expected for STED imaging of thick tissue. However, this will be part of further studies.

## Conclusions

The present study demonstrated the successful upgrade of a conventional confocal fluorescence microscope to perform diffraction-unlimited imaging of biological targets *in vitro* and *in situ*. With the priority laid on cost and time efficiency rather than optimal performance at the level of commercial STED systems, we were able to realize this by just additionally implementing an amplified and frequency-doubled laser diode and some optical components (listed in S1 Table) to the existing confocal microscope. TCSPC detection and evaluation software from the confocal microscope that was the basis for the upgrade did not need alteration and allowed for offline time gating of images taken in the all pulsed modality as implemented here.

Background fluorescence, scattering, and out of focus light may be problematic in the diffraction-unlimited imaging of complex biological tissues. Established deconvolution methods can be easily used to improve the signal-to-background ratio, also of (2D-) STED images. Comparatively, we have applied a recently proposed Richardson-Lucy deconvolution approach and showed that images gained from time-gated pulsed STED microscopy may benefit with regard to the SBR from the joint deconvolution of sub-images with different spatial information that are obtained from offline time gating. Further improvement may be expected in the future by taking a third time gate with time-dependent PSF into account for multi-image deconvolution.

Multicolor imaging for separating different molecular species is an important issue for many biomedical investigations. The simple STED upgrade presented here is already operable for these kinds of studies. The all pulsed modality in combination with the TCSPC detection allows for the separation of at least two dyes by their fluorescence decay times [19]. Thus, fluorescence lifetime imaging microscopy (FLIM) can be used for co-localization studies without modification of the setup, if the labels differ sufficiently in their fluorescence decays [70]. Multicolor STED imaging has also been shown to work with dyes that can be excited with the same excitation laser wavelength and de-excited by the same STED wavelength, but show (subtle) differences in their emission spectra [43].

## Supporting Information

S1 Table List of components. (DOCX)

S1 Fig Test for possible misalignment effects on the PSF shape due to scanning the objective over a range of 80×80 µm^2^. The backscattered light from gold nanoparticles (diameter of 80 nm) was imaged at different positions of the entire field of view accessible to the used piezo scanner (80×80 µm^2^). The upper of the two beads in the center of (A) (dotted box #1) was imaged at a higher resolution (20 nm per pixel), applying a pulse interleave technique that relies on alternating pulsing of the two lasers (635 nm and 766 nm) both at a repetition rate of 40 MHz. TCSPC detection allows the temporal distinction between the scattered light of different wavelengths, as bursts of photons from either laser source are collected with a delay of ~12.5 ns. (B1) shows an overlay of the corresponding quasi simultaneously determined PSFs of the 635 nm excitation and 766 nm depletion light in red and green, respectively. (C1) shows the 766 nm PSF alone for inspection of the central minimum. By moving the microscope stage relative to the central piezo scanner position of the objective, the same gold bead was moved to different positions in the scan field (red boxes #2, #3, #4, and #5 in (A)) and imaged at high spatial resolution. The resulting PSF images are shown in (B) and (C). As the scan range (-40 to + 40 μm) is small compared to the beam diameter of 4 mm (1/e^2^-diameter of spatial power distribution) overfilling the objective’s back aperture by less than 1%, no effects of misalignment (B) or deformation of the doughnut (C) are visible. (D) Profile plots through the intensity minima of (C) confirm that effects of the displacement during scanning on the PSF are small. Decreasing intensity toward the periphery of the scan range cannot be observed.
(TIFF)

S2 Fig Effective point spread function (PSF). The lateral intensity distributions of 12 single crimson beads (20 nm) imaged in the STED mode were averaged to determine the effective PSF shape of the system (red dots). The upper graph shows a least-square fit of a 2D-Lorentzian function to the averaged intensity data. The lower graph shows a Gaussian-2D function fitted to the same data. The Gaussian function did not describe the PSF precisely, particularly the maximum at the peak’s center showed a strong deviation between simulation and experimental data. Blue and black points represent the projections of the data to the *xz*- and *yz*-planes.
(TIFF)

S3 Fig Influence of relative intensity scaling of the sub-images merged by the multi-image deconvolution on the reconstruction results. Images with different spatial information extracted from offline time gating of a pulsed STED imaging run (S5 Fig) were jointly deconvolved (B-D) and compared to a conventional Richardson-Lucy deconvolution of the STED image (late photon gate) alone (A). To have a comparable stopping criterion for the iterative algorithm, we monitored the width of a characteristic feature in the resulting image as a function of the number of iterations. Here, we chose the intensity distribution along the yellow line in (A) that apparently stems from a single actin filament (with a diameter < 20 nm). Corresponding profile plots of chosen iteration steps are shown in the right column. Specified FWHM values were determined by simulation of the data (symbols) with Gaussian functions (lines). The algorithm was stopped when the FWHM width reached a value of 50 nm, that is, 2.5 times the pixel size of 20 nm used in the image acquisition. As PSFs for the deconvolutions resulting in (A–D), a 2D-Lorentzian peak function with FWHM of 70 nm and a 2D-Gaussian (FWHM 300 nm) were applied for the STED and the confocal image respectively, as determined in S5 Fig. While in the joint deconvolution represented by (B) the two input sub-images were processed without any intensity scaling, in (C) all pixel values of the confocal sub-image were scaled by a factor of 2 and in (D) by a factor of 4 before starting the RL algorithm. The number of iterations that were necessary to reach the anticipated width of 50 nm in the image of a single filament rose with the increasing importance of the confocal image. Changes in the deconvolved image that correlate with the scaling factor are discernible at the position marked by the white arrow (in A). In the deconvolved STED image (A), only three relatively dark spots remain as fluorescence signals from the filament. From the raw image (S5A Fig), it becomes obvious that the signal in this region is very low, resulting in a bad signal-to-noise ratio. At this special position, the extra signal from the confocal image, when included into a joint deconvolution, increases the relative brightness of these spots (B). By further increasing the importance of the confocal sub-image, the spottiness of the deconvolved image of the single marked filament is further reduced. We note that we cannot exclude completely that the ‘spotty’ solution of the algorithm may reveal the correct distribution of emitting fluorescent molecules. But, we consider it implausible that the collected fluorescence at the discussed region of the filament stems from molecules located precisely at the spots appearing in the deconvolved image. We rather regard this as an artifact resulting from the extremely poor SNR at this position of the image. A common way to counter noise amplification is to include constraints based on a-priori knowledge of the structure to exclude solutions to the ill-posed problem of image restoration by deconvolution. The addition of the information of the confocal image in the joint approach can be interpreted as such a constraint on the solution of the deconvolution, with the scaling factor being the related regularization parameter.
(TIFF)

S4 Fig Richardson-Lucy deconvolution of immunofluorescence localization of microtubules in blowfly salivary glands using the dye STAR635P. The resulting reconstructed image of a conventional RL deconvolution of a time gated STED image (A) is compared to that of a joint multi-image deconvolution (B) of two sub-images with different spatial information won by offline time gating the data of one single pulsed STED image acquisition run (see Fig 6). The white box indicates a feature whose diameter was imaged as a function of calculated iteration steps (right column) and served to define a comparable stopping criterion. Analogous to the image deconvolution of actin filaments (S3 Fig), the algorithm was stopped when a diameter of 2.5 times the pixel size (of the raw images) was achieved. With a pixel size of 25 nm, this lead to a diameter of 62.5 nm as a stopping criterion, which was achieved after 16 iterations in the case of the single STED image deconvolution (A) and after 131 iterations in the case of the joint two-image deconvolution (B). The PSFs were chosen to be a 2D-Lorentzian peak function (FWHM = 70 nm) and a 2D-Gaussian (FWHM = 300 nm), like before. A weighting of the two raw images was not applied, as the choice of an optimal value for this additional parameter has not been investigated. A comparison of images (A) and (B) shows that, in the result of the joint deconvolution, the SBR is further enhanced relative to the SBR in the single-image deconvolution. Here, we understand the bright strands in the image as signals from the labeled microtubules, and the rather featureless and somewhat darker intensity distribution as the background. As a local estimation of the SBR, the profile plots (right column) across one single strand can be evaluated for a more quantitative statement. If the amplitude of the peak functions that were used to simulate the cross section intensities are taken as a measure of the signal and the offset of the peaks is taken as a measure of the local background we conclude the SBR to be 1.4 for the raw STED image, 4.1 for the simple deconvolution and 5.8 for the joint deconvolution.
(TIFF)

S5 Fig Estimation of the widths of the point spread functions as parameters for the joint deconvolution of actin filament images. Intensity profiles along neighboring horizontal pixel lines perpendicular to a filament (white boxes in (A) and (B)) were averaged in the STED and the confocal image, respectively. (C) The resulting profiles were simulated with a Lorentzian function for the STED image and a Gaussian function for the confocal image. The models describe the data well and the FWHM values resulting from the simulation are indicated in the graph. From the relative low brightness (compared to regions of the image in which several filaments cross) and its isolated position (from other filamentous structures), it can be concluded that at the chosen position (white box) a single filament is observed. Having a diameter of only 7 nm [63] and being stained with small phalloidin conjugates, the fluorescence labelled actin filament should have a diameter well below 20 nm, and the fluorescence intensity profile in (C) reveals the resolving power of the optical system not heavily influenced by the real diameter of the structure. From Crimson beads (diameter of 20 nm), a radial symmetry of the effective PSF of our STED setup was derived (S2 Fig). For the deconvolution of STED images of F-actin we chose symmetric 2D-Lorentzian (STED) and 2D-Gaussian (confocal) peak functions with a diameter determined as a cross-section of a single filament from the images to be deconvolved. STED imaging of F-actin labeled with STAR635-phalloidin (A) with an average depletion power of 5 mW at a repetition rate of 2.5 MHz. See (S7–S9) for corresponding deconvolution results.
(TIFF)

S6 Fig Comparison of the resolving power of the STED upgrade at laser repetition rates of 2.5 MHz and 40 MHz in imaging Crimson beads. Crimson beads embedded in Mowiol were imaged in gated STED mode under identical experimental settings (pixel dwell time 1 ms, pixel size 20 nm) except for the repetition rate of the lasers that was changed from 2.5 MHz (A) to 40 MHz (B). The mean STED power of 90 mW at 40 MHz corresponded to a mean power of 10 mW at 2.5 MHz, resulting in a higher per pulse energy at the 16 times lower repetition rate. Accordingly, the resolving power was higher at 2.5 MHz as the mean values of FWHM values of single peaks in (A) and (B) indicate. Arrows in (A) and (C) mark peaks that were simulated by means of least square fitting a Lorentzian function (B and D) to determine FWHM values. (E) Statistic box plots where the mean value is given as open square, the box indicates the interquartile range, and the thick line in the box represents the median value. The error bars give the standard deviation and outliers are plotted as diamond shaped symbols. As expected due to the higher pulse energy accessible by our STED laser at lower repetition rates, the spatial resolution determined as the FWHM of the intensity peaks of Crimson beads in the STED image, seems to be higher at 2.5 MHz.
(TIFF)

S7 Fig Results of a Richardson-Lucy deconvolution of the time gated STED image at different iteration steps. The raw STED image assembled by electing only counts in the third time gate (Fig 2A) was deconvolved via application of the RL algorithm using a Lorentzian PSF with FWHM diameter of 70 nm. Reconstructed images after 1, 10, 30, 100, and 300 iterations are shown. The insets (lower left corner) give the power spectra of the discrete Fourier transform (performed using Fiji [71]) of each image. Obviously, there are higher frequencies present in the raw STED image compared to the confocal image (S8 Fig). Amplification of the high frequencies of the RL algorithm thus requires a lower number of iterations. As a result (and probably also due to a lower signal-to-noise ratio), amplification of noise visible as ‘spottiness’ in the reconstructed image becomes an issue already after a few iterations in the darker regions and after some tens of iterations in the brighter structures.
(TIFF)

S8 Fig Results of a Richardson-Lucy deconvolution of the confocal ‘early time gate’ image at different iteration steps. The raw confocal image assembled by electing only counts in the first time gate (Fig 2A) was deconvolved via application of the RL algorithm using a Gaussian PSF with FWHM diameter of 300 nm. (Pixel size of raw image and deconvolved images: 20 nm). Reconstructed images after 1, 10, 100, 1000, and 10000 iterations are shown. The insets (lower left corner) give the power spectra of the discrete Fourier transform (performed using Fiji [71]) of each image. It can be seen qualitatively how noise is suppressed by blurring with the Gaussian PSF in the first step and how the high frequencies are recovered with an increasing number of iterations. Depending on the relative intensity of features, ‘spottiness’ as a result of noise amplification becomes visible after ~100 iterations in the darker part of the image and after ~1000 iterations in the brighter parts.
(TIFF)

S9 Fig Results of a Richardson-Lucy deconvolution for the joint processing of images with different spatial information gained through offline time gating at different iteration steps. The sum of the raw STED image (counts in the third time gate) and the raw confocal image (counts in the first time gate) is shown together with the power spectrum of the discrete Fourier transform (inset), see Fig 4 (left column). Joint deconvolution of the raw images was performed as described by the application of the RL algorithm using a Lorentzian PSF with a FWHM diameter of 70 nm and a Gaussian PSF with a FWHM diameter of 300 nm. Reconstructed images after 1, 10, 30, 100, and 300 iterations are shown. Higher frequencies are recovered faster (less iterations) than in the case of confocal deconvolution only (S8 Fig). Compared to the STED-only deconvolution (S7 Fig), more iterations are required to reconstruct the high frequencies in the image. For a detailed comparison of the joint multi-image deconvolution with the deconvolution of the STED image alone, see S3 Fig.
(TIFF)

S10 Fig Joint deconvolution of fluorescence images of F-actin filaments stained with Atto647N-phalloidin acquired in gated STED mode. (A) Diffraction-limited and-unlimited images of a gated STED acquisition of a filament structure attached to the glass cover slip and labeled with the dye Atto647N. STED power of 5 mW at 2.5 MHz repetition rate. In the bottom row, the marked region of the STED image is shown enlarged and compared to the results of a Richardson-Lucy deconvolution of the STED image (STED dec.), and the result of a joint deconvolution (joint dec.). At the position marked by the white triangle two adjacent filaments may be assumed in the raw image, while in the jointly deconvolved image this feature appears suppressed stronger than in the case of the conventional deconvolution. This demonstrates that through the relative reduction of background and noise in the deconvolved image also badly defined features at low brightness may be suppressed. Parameter for the conventional deconvolution: 12 iterations with a Lorentzian functions of width 60 nm (determined from Fig 5C); 49 Iterations with Lorentzian function (60 nm) and Gaussian (300 nm) weighting input images by 1:1.
(TIFF)

S11 Fig F-actin filaments in blowfly salivary glands. Gland cryosections (10 µm thick) were labeled with the F-actin probe Abberior STAR635-phalloidin. (A-C) A confocal overview image as well as enlarged views of regions indicated as yellow rectangles are shown. The basal region of the secretory cells showed weak fluorescence (thick arrow) indicating only marginal infoldings compared to the apical membrane [72,73]. In contrast, the canaliculi appeared as brightly fluorescent structures with widths in the micrometer range, indicating F-actin filament bundles lining the canaliculi (thin arrow). (D) STED image of the region shown in (C). TEM studies have unraveled further infolding of the canaliculi forming densely packed sheet like microvilli (so-called microplicae) with a microplicae having a thickness of 60–80 nm, while exhibiting variable lengths and widths. In addition, the microplicae are closely packed forming a fine extracellular space of approx. 10–20 nm between two adjacent microplicae [69]. Thus, this microplicae arrangement could represent an appropriable target for STED microscopy. However, no substructures of the canaliculi could be observed in confocal images (B-C). In the corresponding STED image (D) substructures within the canaliculus could be assumed, which were blurred out in the corresponding confocal image in (C). Nevertheless, the dense microplicae arrangement and thus the high density of F-actin seems to be hindering STED imaging, especially with diffraction-limited resolution in *z*-direction. The dense F-actin packing most probably influences the labeling efficiency, complicating the access of dye molecules to different parts of the microplicae. Thus, the labeled structure could differ from the defacto F-actin filament bundles, resulting in a heterogeneous fluorescence signal distribution as can be seen in (D).
(TIFF)
