# Supplementary material for: Upgrade of a Scanning Confocal Microscope to a Single-Beam Path STED Microscope
Source: PLoS One. 2015 Jun 19;10(6):e0130717. doi: 10.1371/journal.pone.0130717 (PMC4475078; doi:10.1371/journal.pone.0130717)
Supplement: S1 Table — (DOCX) [file pone.0130717.s001.docx]

**S1 Table. List of components necessary for the upgrade of the confocal scanning microscope.** We assume the confocal scanning microscope to be upgraded is already equipped with a ~640 nm excitation laser source, the corresponding single-mode fiber coupling and collimation components, a high-performance plan apochromatic objective, and a time-correlated single-photon counting (TCSPC) module.

| **Component** | **Quantity** | **Function** | **Our choice** | **Manufacturers/ distributors of comparable components** |
| --- | --- | --- | --- | --- |
| High power ps-pulsed NIR laser | 1 | Delivers STED pulses | LDH-P-FA-765, PicoQuant | Onefive GmbH,  MPB Communications Inc. |
| NIR mirror | 2 | STED beam steering | BB1-E03, Thorlabs | Optics distributors^1^ |
| Kinematic mirror mount | 2 | STED beam steering for single mode fiber coupling | KS1, Thorlabs | Optomechanics distributors^1^ |
| Longpass dichroic beamsplitter | 1 | Combine excitation and STED beam for joint coupling into the single mode fiber | H 643 LPXR, AHF Analysentechnik | Omega Optical, Chroma,  Semrock |
| Kinematic filter holder | 1 | Excitation beam steering | H45 in combination with KS1, Thorlabs | Optomechanics distributors^1^ |
| End-Cap PM fiber | 1 | Withstand of high STED laser power | P-FAnskFUnsk-4/125/5s-3 630PM; Coastal Connections | NKT photonics,  Thorlabs |
| Fiber connector adapter | 1 | Flange mount for the fiber | 10AF-0-FC, Schäfter+Kirchhoff | Optomechanics distributors^1^ |
| High extinction rate Polarizer | 1 | Clean linear polarization state of STED light | Glan-Thompson polarizer 50.230.00010, Artifex-Engineering | Bernhard Halle Nachfl.,  Optics distributors^1^ |
| Achromatic quarter wave plate | 1 | Change laser light to circular polarization | Achrom. (500-800 nm) wave plate, 50.260.00198, Artifex-Engineering | Bernhard Halle Nachfl.,  Optics distributors^1^ |
| (Precision) Rotation mount | 1 | Orientation adjustment of quarter wave plate | PRM1/M, Thorlabs | Optomechanics distributors^1^ |
| Dual line beamsplitter | 1 | Separation of excitation and fluorescence | Zt 635/766 rpc, AHF Analysentechnik | Omega Optical, Chroma, Semrock |
| Segmented phase plate | 1 | Create STED doughnut | EASYDOnut Phaseplate 640/765, Abberior | -- |
| XY translation mount | 1 | Lateral alignment of phase plate | Inhouse made holder, alternatively a Centering Mounting Holder G06102500, Qioptiq, may be useable | Optomechanics distributors^1^ |
| Bandpass emission filter | 1 | Barrier filter in front of the detector | 690/70 H Bandpass, AHF Analysentechnik | Omega Optical, Chroma, Semrock |

^1^ Optics/Optomechanics manufacturers/distributors: e.g. Laser2000, Newport, Qioptiq, Owis, Edmund Optics, Thorlabs, OptoSigma
