# Supplementary figures and images for: Upgrade of a Scanning Confocal Microscope to a Single-Beam Path STED Microscope
Source: PLoS One. 2015 Jun 19;10(6):e0130717. doi: 10.1371/journal.pone.0130717 (PMC4475078; doi:10.1371/journal.pone.0130717)

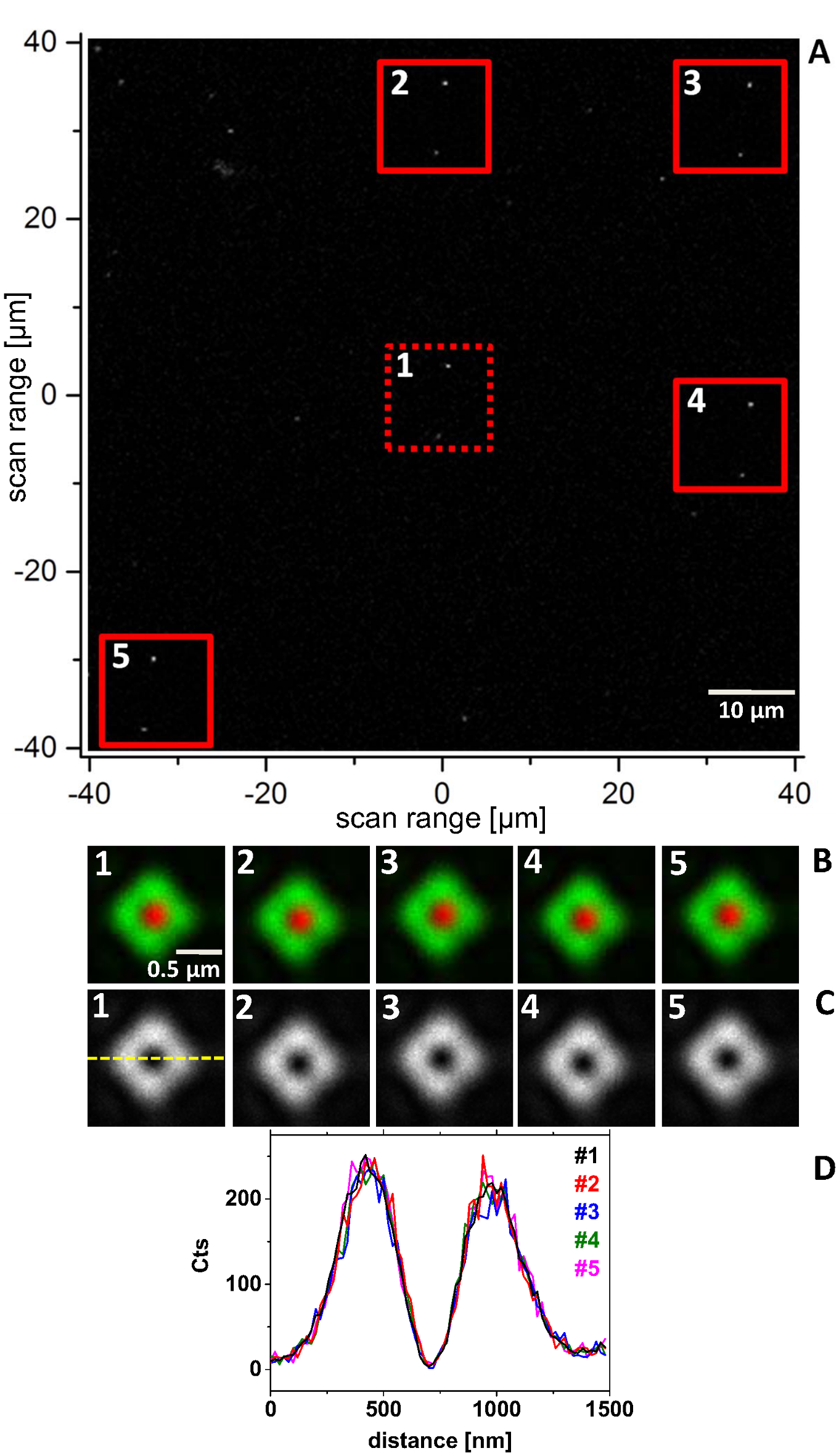

Supplement: S1 Fig — The backscattered light from gold nanoparticles (diameter of 80 nm) was imaged at different positions of the entire field of view accessible to the used piezo scanner (80×80 µm2). The upper of the two beads in the center of (A) (dotted box #1) was imaged at a higher resolution (20 nm per pixel), applying a pulse interleave technique that relies on alternating pulsing of the two lasers (635 nm and 766 nm) both at a repetition rate of 40 MHz. TCSPC detection allows the temporal distinction between the scattered light of different wavelengths, as bursts of photons from either laser source are collected with a delay of ~12.5 ns. (B1) shows an overlay of the corresponding quasi simultaneously determined PSFs of the 635 nm excitation and 766 nm depletion light in red and green, respectively. (C1) shows the 766 nm PSF alone for inspection of the central minimum. By moving the microscope stage relative to the central piezo scanner position of the objective, the same gold bead was moved to different positions in the scan field (red boxes #2, #3, #4, and #5 in (A)) and imaged at high spatial resolution. The resulting PSF images are shown in (B) and (C). As the scan range (-40 to + 40 μm) is small compared to the beam diameter of 4 mm (1/e2-diameter of spatial power distribution) overfilling the objective’s back aperture by less than 1%, no effects of misalignment (B) or deformation of the doughnut (C) are visible. (D) Profile plots through the intensity minima of (C) confirm that effects of the displacement during scanning on the PSF are small. Decreasing intensity toward the periphery of the scan range cannot be observed. (TIFF) [file pone.0130717.s002.tiff]

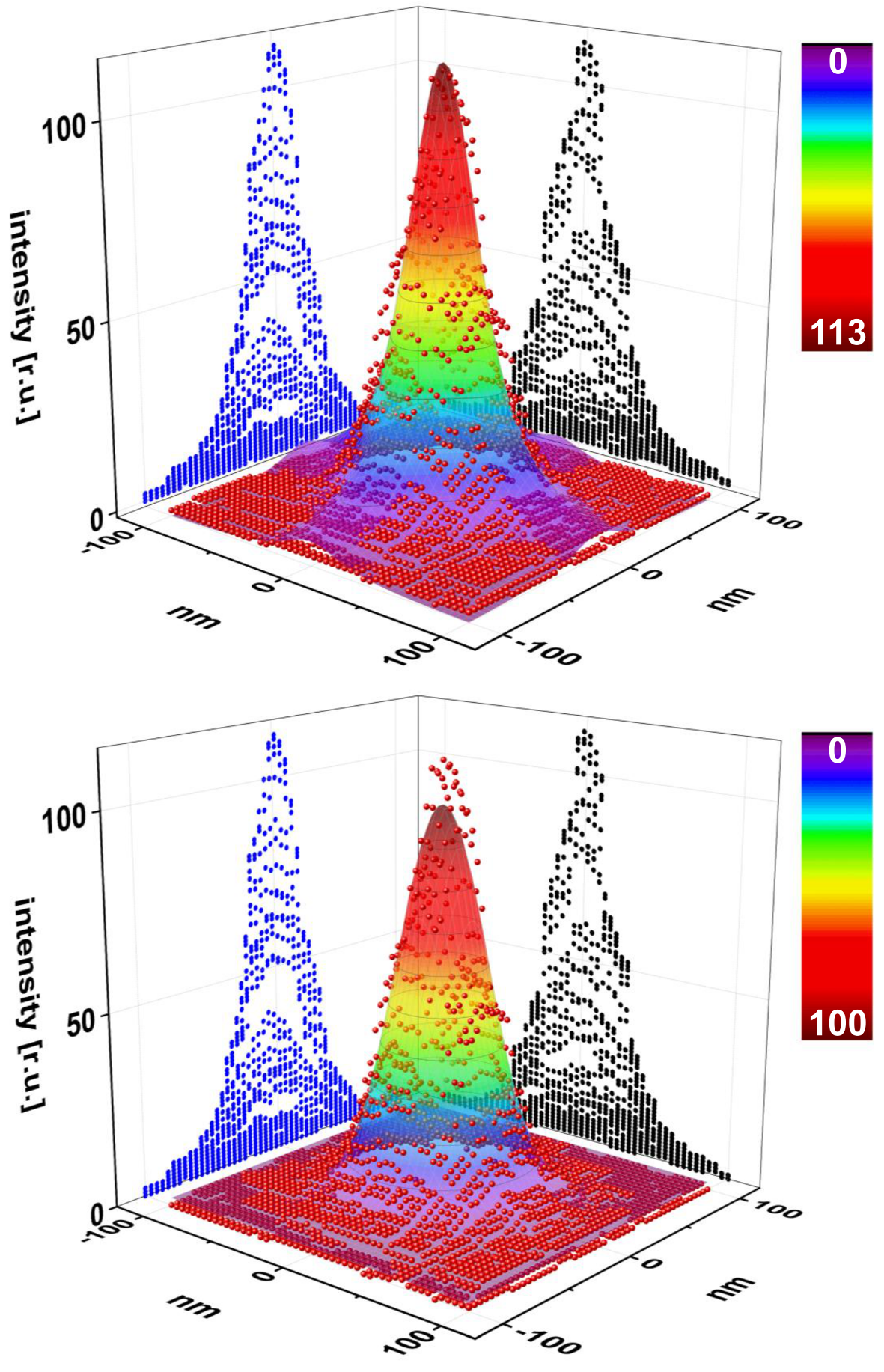

Supplement: S2 Fig — The lateral intensity distributions of 12 single crimson beads (20 nm) imaged in the STED mode were averaged to determine the effective PSF shape of the system (red dots). The upper graph shows a least-square fit of a 2D-Lorentzian function to the averaged intensity data. The lower graph shows a Gaussian-2D function fitted to the same data. The Gaussian function did not describe the PSF precisely, particularly the maximum at the peak’s center showed a strong deviation between simulation and experimental data. Blue and black points represent the projections of the data to the xz- and yz-planes. (TIFF) [file pone.0130717.s003.tiff]

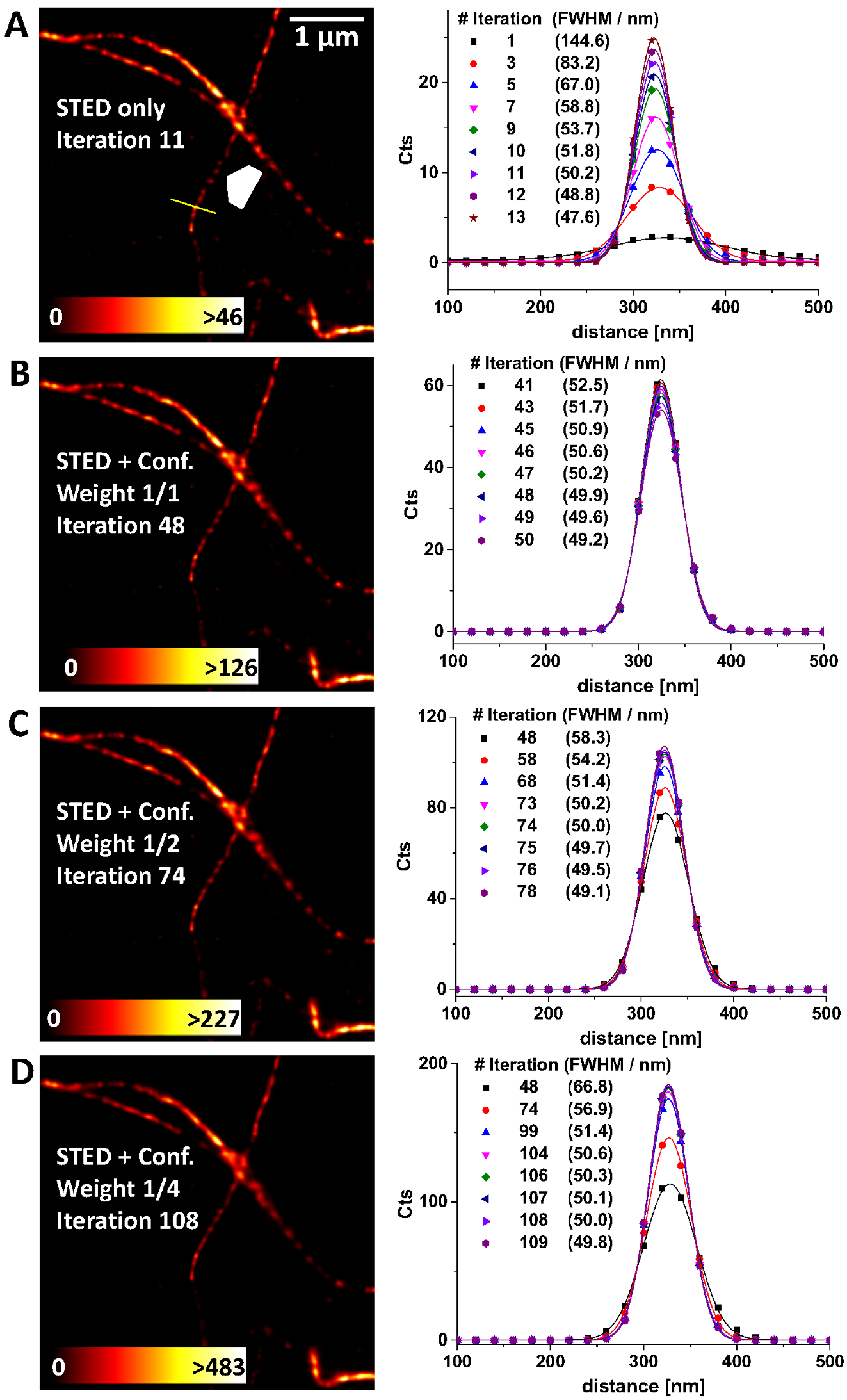

Supplement: S3 Fig — Images with different spatial information extracted from offline time gating of a pulsed STED imaging run (S5 Fig) were jointly deconvolved (B-D) and compared to a conventional Richardson-Lucy deconvolution of the STED image (late photon gate) alone (A). To have a comparable stopping criterion for the iterative algorithm, we monitored the width of a characteristic feature in the resulting image as a function of the number of iterations. Here, we chose the intensity distribution along the yellow line in (A) that apparently stems from a single actin filament (with a diameter < 20 nm). Corresponding profile plots of chosen iteration steps are shown in the right column. Specified FWHM values were determined by simulation of the data (symbols) with Gaussian functions (lines). The algorithm was stopped when the FWHM width reached a value of 50 nm, that is, 2.5 times the pixel size of 20 nm used in the image acquisition. As PSFs for the deconvolutions resulting in (A–D), a 2D-Lorentzian peak function with FWHM of 70 nm and a 2D-Gaussian (FWHM 300 nm) were applied for the STED and the confocal image respectively, as determined in S5 Fig. While in the joint deconvolution represented by (B) the two input sub-images were processed without any intensity scaling, in (C) all pixel values of the confocal sub-image were scaled by a factor of 2 and in (D) by a factor of 4 before starting the RL algorithm. The number of iterations that were necessary to reach the anticipated width of 50 nm in the image of a single filament rose with the increasing importance of the confocal image. Changes in the deconvolved image that correlate with the scaling factor are discernible at the position marked by the white arrow (in A). In the deconvolved STED image (A), only three relatively dark spots remain as fluorescence signals from the filament. From the raw image (S5A Fig), it becomes obvious that the signal in this region is very low, resulting in a bad signal-to-noise ratio. At this special pos [file pone.0130717.s004.tiff]

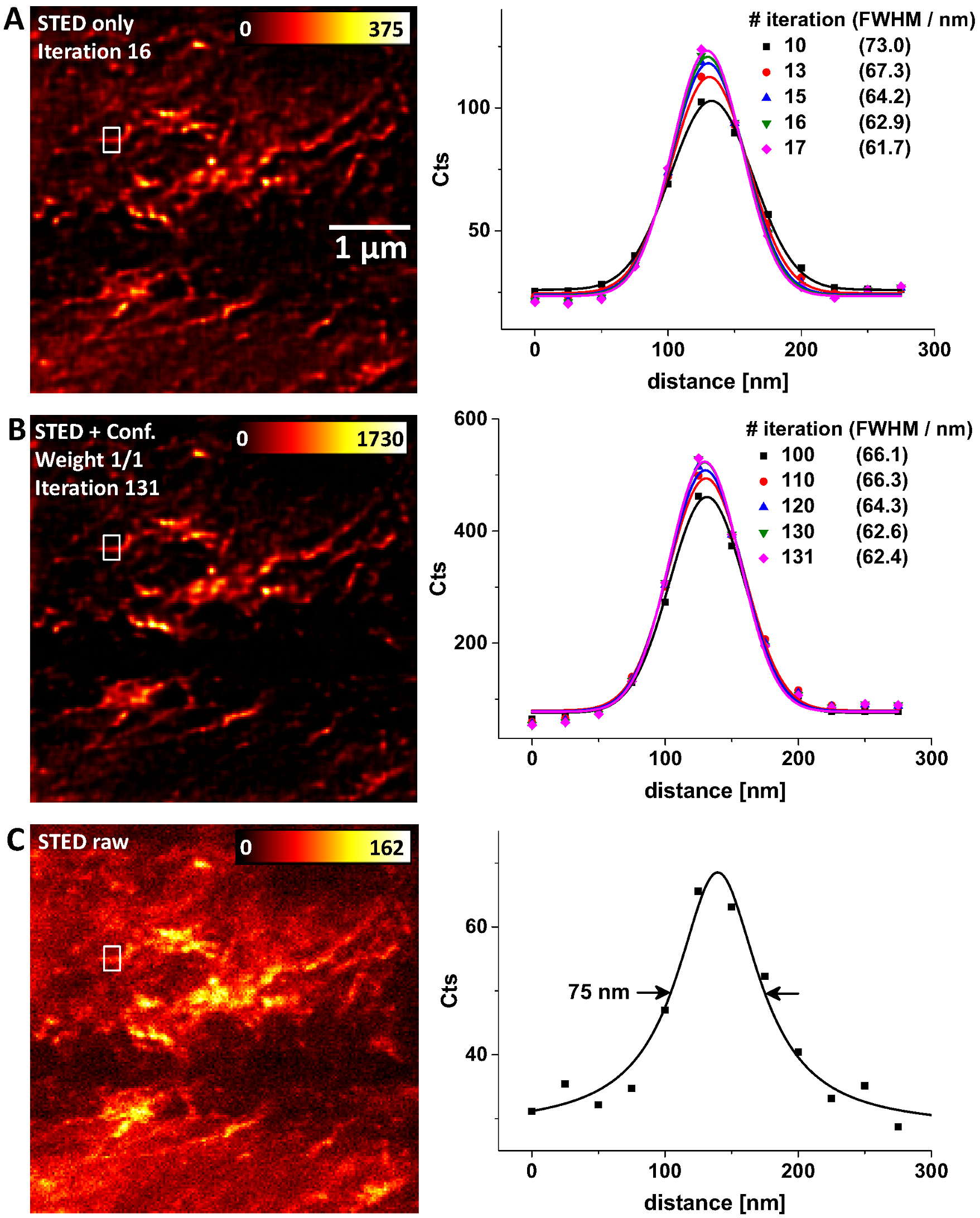

Supplement: S4 Fig — The resulting reconstructed image of a conventional RL deconvolution of a time gated STED image (A) is compared to that of a joint multi-image deconvolution (B) of two sub-images with different spatial information won by offline time gating the data of one single pulsed STED image acquisition run (see Fig 6). The white box indicates a feature whose diameter was imaged as a function of calculated iteration steps (right column) and served to define a comparable stopping criterion. Analogous to the image deconvolution of actin filaments (S3 Fig), the algorithm was stopped when a diameter of 2.5 times the pixel size (of the raw images) was achieved. With a pixel size of 25 nm, this lead to a diameter of 62.5 nm as a stopping criterion, which was achieved after 16 iterations in the case of the single STED image deconvolution (A) and after 131 iterations in the case of the joint two-image deconvolution (B). The PSFs were chosen to be a 2D-Lorentzian peak function (FWHM = 70 nm) and a 2D-Gaussian (FWHM = 300 nm), like before. A weighting of the two raw images was not applied, as the choice of an optimal value for this additional parameter has not been investigated. A comparison of images (A) and (B) shows that, in the result of the joint deconvolution, the SBR is further enhanced relative to the SBR in the single-image deconvolution. Here, we understand the bright strands in the image as signals from the labeled microtubules, and the rather featureless and somewhat darker intensity distribution as the background. As a local estimation of the SBR, the profile plots (right column) across one single strand can be evaluated for a more quantitative statement. If the amplitude of the peak functions that were used to simulate the cross section intensities are taken as a measure of the signal and the offset of the peaks is taken as a measure of the local background we conclude the SBR to be 1.4 for the raw STED image, 4.1 for the simple deconvolution and 5.8 for the joint deconvol [file pone.0130717.s005.tiff]

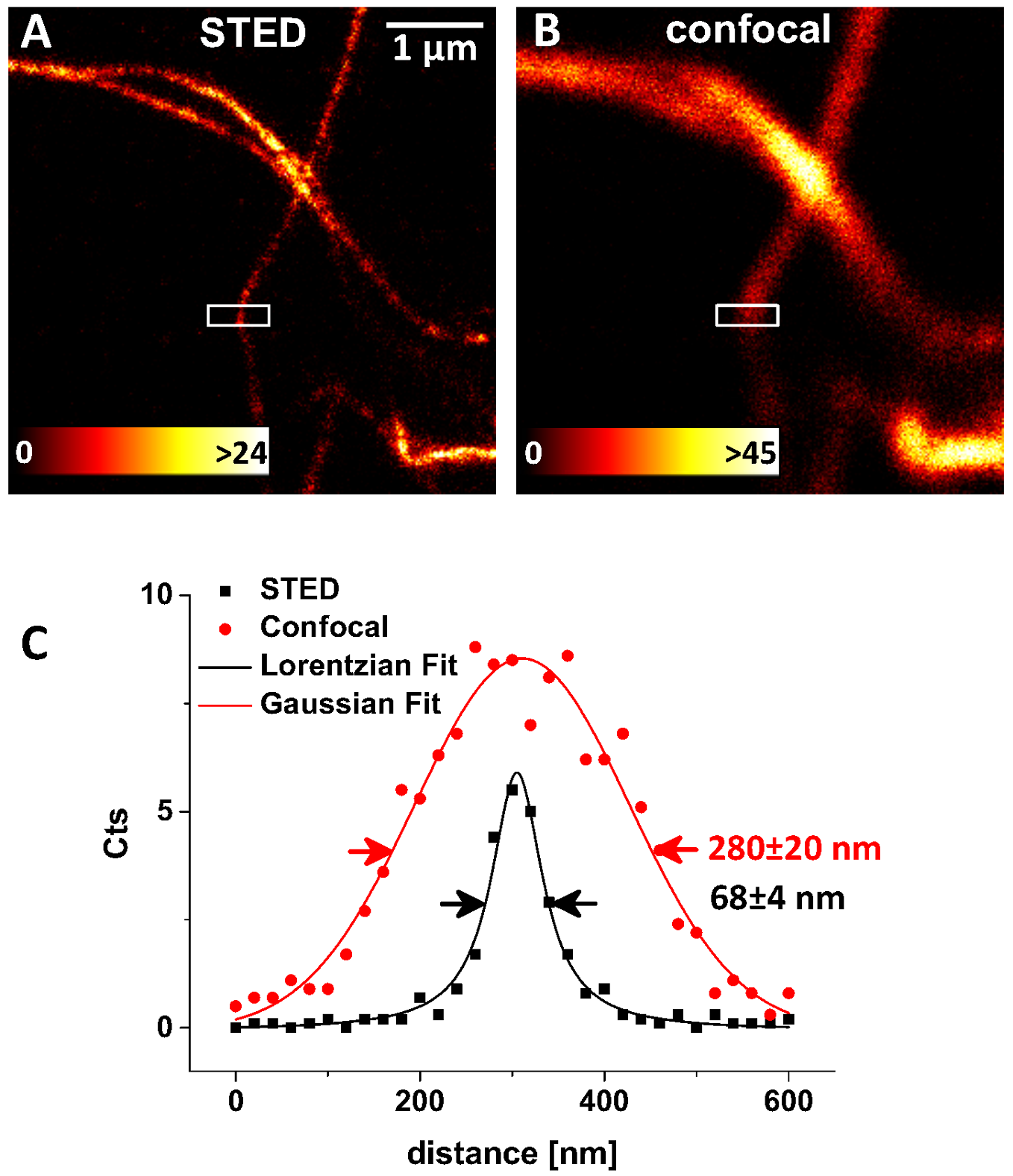

Supplement: S5 Fig — Intensity profiles along neighboring horizontal pixel lines perpendicular to a filament (white boxes in (A) and (B)) were averaged in the STED and the confocal image, respectively. (C) The resulting profiles were simulated with a Lorentzian function for the STED image and a Gaussian function for the confocal image. The models describe the data well and the FWHM values resulting from the simulation are indicated in the graph. From the relative low brightness (compared to regions of the image in which several filaments cross) and its isolated position (from other filamentous structures), it can be concluded that at the chosen position (white box) a single filament is observed. Having a diameter of only 7 nm [63] and being stained with small phalloidin conjugates, the fluorescence labelled actin filament should have a diameter well below 20 nm, and the fluorescence intensity profile in (C) reveals the resolving power of the optical system not heavily influenced by the real diameter of the structure. From Crimson beads (diameter of 20 nm), a radial symmetry of the effective PSF of our STED setup was derived (S2 Fig). For the deconvolution of STED images of F-actin we chose symmetric 2D-Lorentzian (STED) and 2D-Gaussian (confocal) peak functions with a diameter determined as a cross-section of a single filament from the images to be deconvolved. STED imaging of F-actin labeled with STAR635-phalloidin (A) with an average depletion power of 5 mW at a repetition rate of 2.5 MHz. See (S7–S9) for corresponding deconvolution results. (TIFF) [file pone.0130717.s006.tiff]

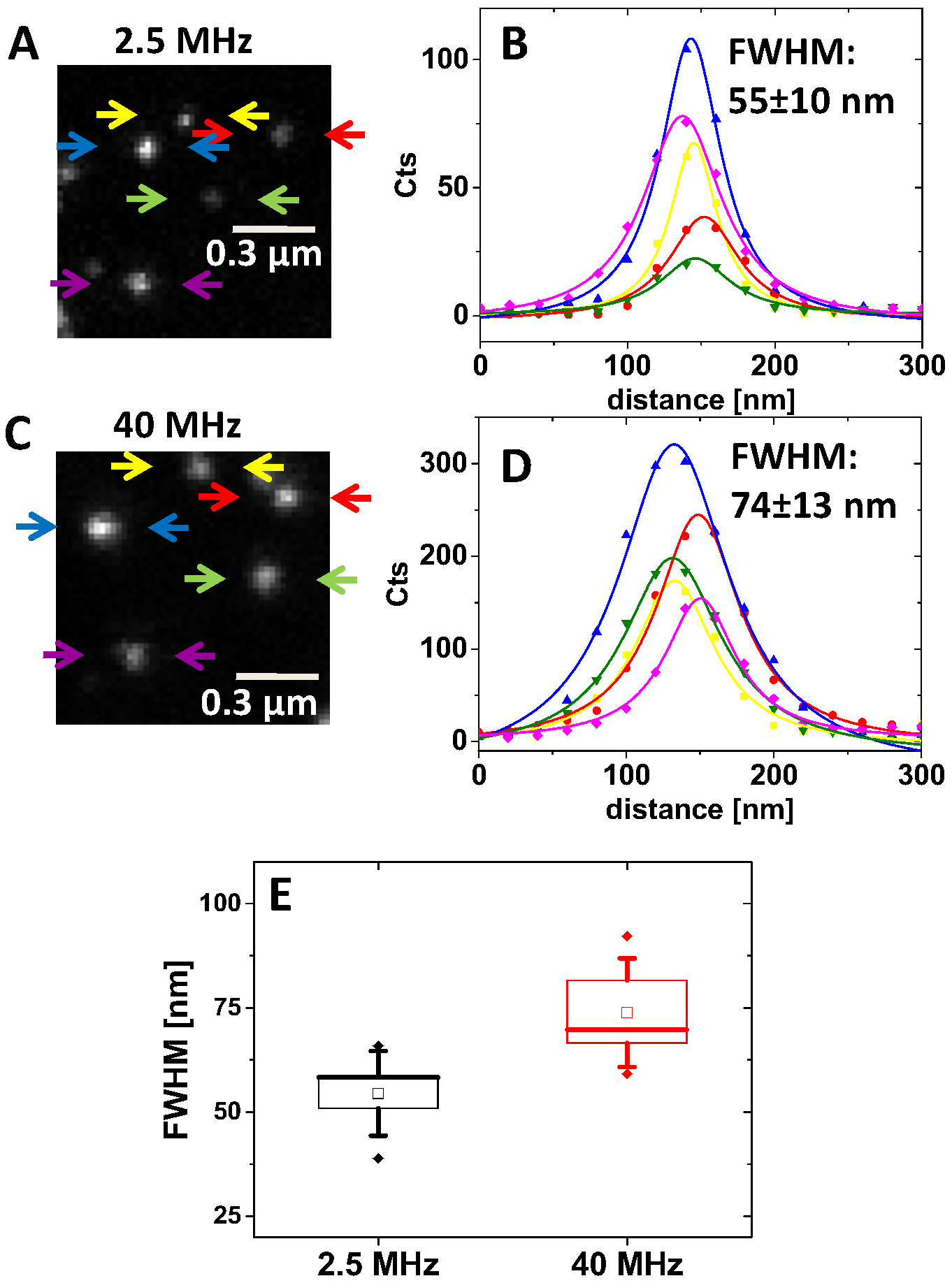

Supplement: S6 Fig — Crimson beads embedded in Mowiol were imaged in gated STED mode under identical experimental settings (pixel dwell time 1 ms, pixel size 20 nm) except for the repetition rate of the lasers that was changed from 2.5 MHz (A) to 40 MHz (B). The mean STED power of 90 mW at 40 MHz corresponded to a mean power of 10 mW at 2.5 MHz, resulting in a higher per pulse energy at the 16 times lower repetition rate. Accordingly, the resolving power was higher at 2.5 MHz as the mean values of FWHM values of single peaks in (A) and (B) indicate. Arrows in (A) and (C) mark peaks that were simulated by means of least square fitting a Lorentzian function (B and D) to determine FWHM values. (E) Statistic box plots where the mean value is given as open square, the box indicates the interquartile range, and the thick line in the box represents the median value. The error bars give the standard deviation and outliers are plotted as diamond shaped symbols. As expected due to the higher pulse energy accessible by our STED laser at lower repetition rates, the spatial resolution determined as the FWHM of the intensity peaks of Crimson beads in the STED image, seems to be higher at 2.5 MHz. (TIFF) [file pone.0130717.s007.tiff]

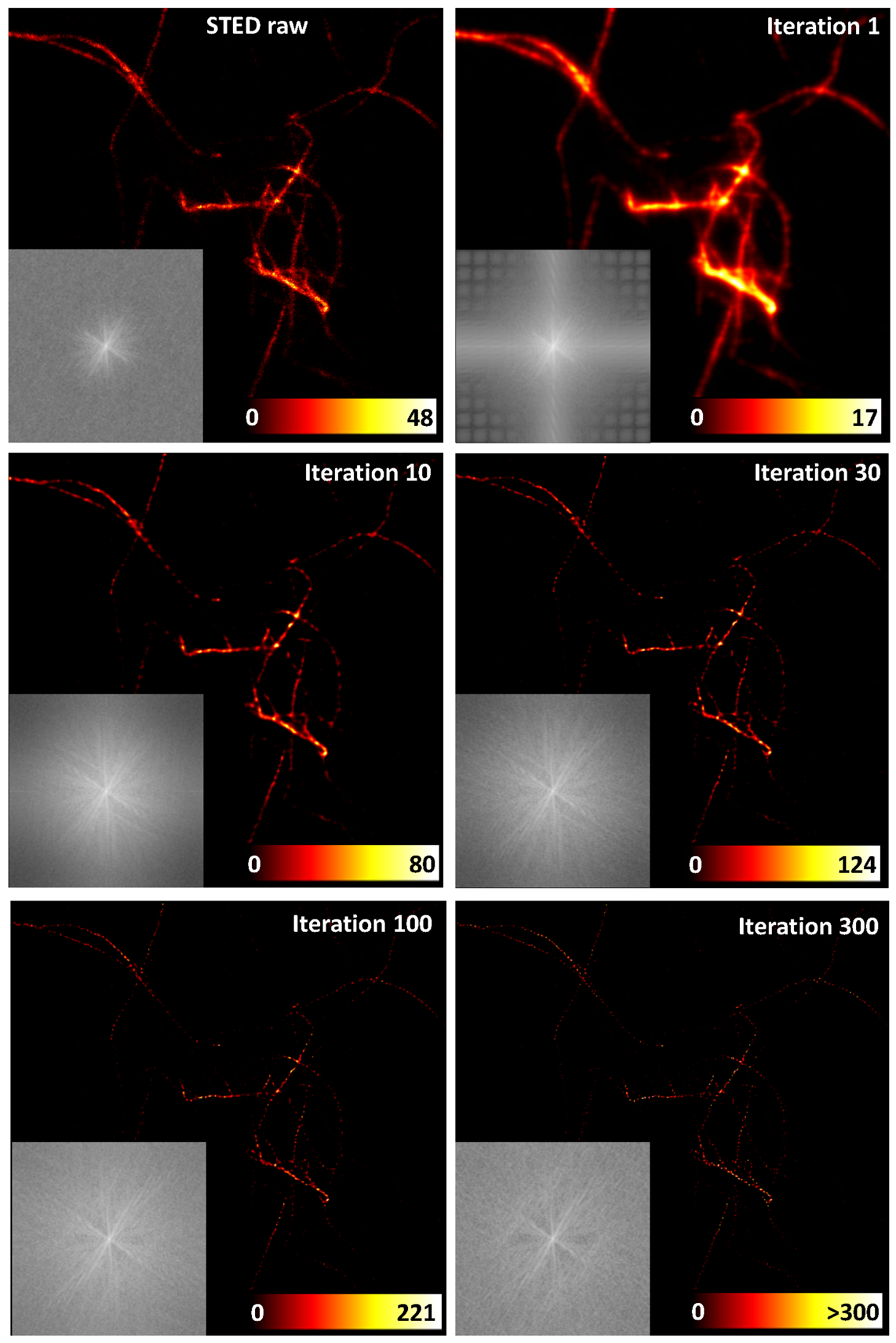

Supplement: S7 Fig — The raw STED image assembled by electing only counts in the third time gate (Fig 2A) was deconvolved via application of the RL algorithm using a Lorentzian PSF with FWHM diameter of 70 nm. Reconstructed images after 1, 10, 30, 100, and 300 iterations are shown. The insets (lower left corner) give the power spectra of the discrete Fourier transform (performed using Fiji [71]) of each image. Obviously, there are higher frequencies present in the raw STED image compared to the confocal image (S8 Fig). Amplification of the high frequencies of the RL algorithm thus requires a lower number of iterations. As a result (and probably also due to a lower signal-to-noise ratio), amplification of noise visible as ‘spottiness’ in the reconstructed image becomes an issue already after a few iterations in the darker regions and after some tens of iterations in the brighter structures. (TIFF) [file pone.0130717.s008.tiff]

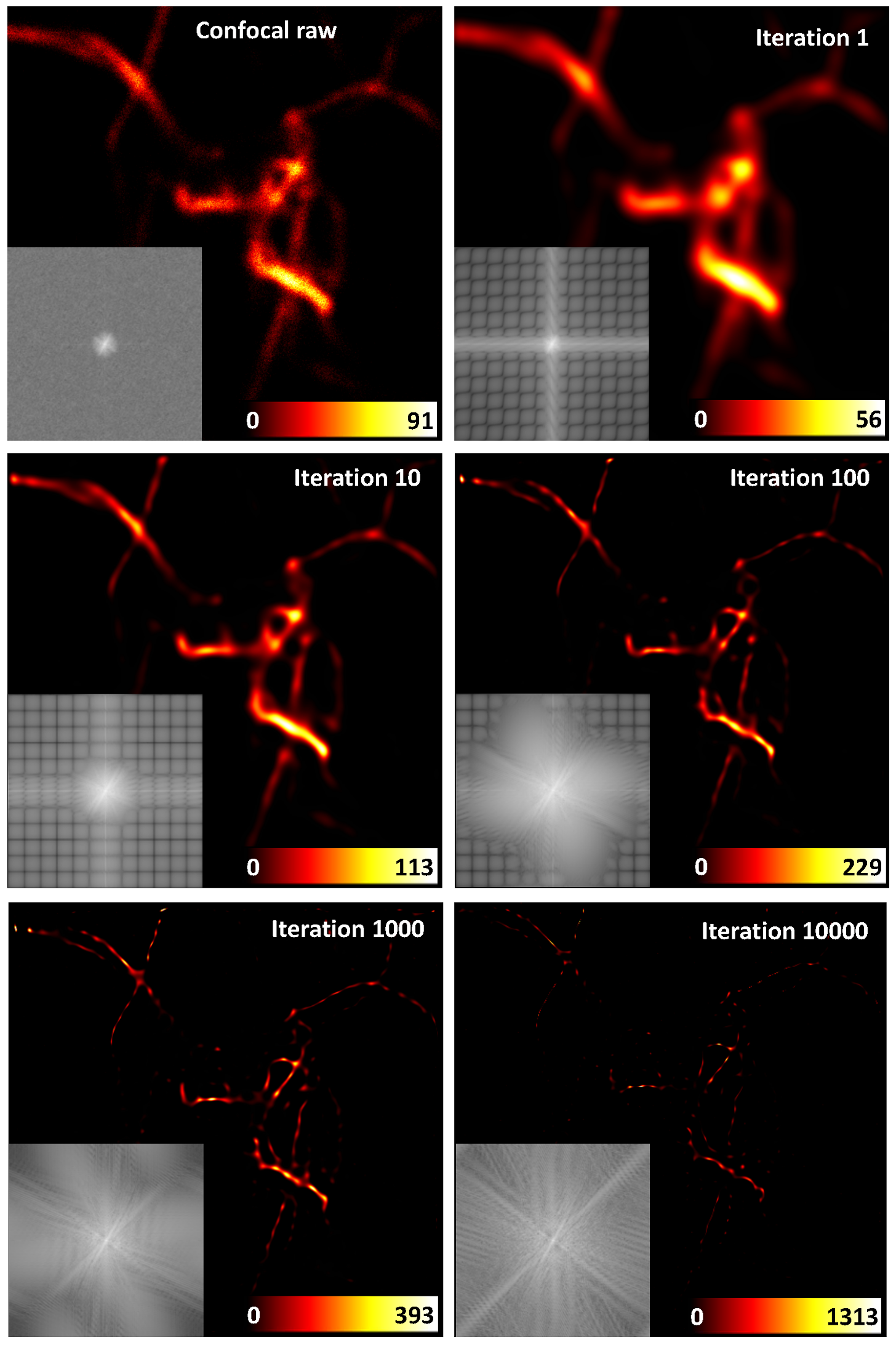

Supplement: S8 Fig — The raw confocal image assembled by electing only counts in the first time gate (Fig 2A) was deconvolved via application of the RL algorithm using a Gaussian PSF with FWHM diameter of 300 nm. (Pixel size of raw image and deconvolved images: 20 nm). Reconstructed images after 1, 10, 100, 1000, and 10000 iterations are shown. The insets (lower left corner) give the power spectra of the discrete Fourier transform (performed using Fiji [71]) of each image. It can be seen qualitatively how noise is suppressed by blurring with the Gaussian PSF in the first step and how the high frequencies are recovered with an increasing number of iterations. Depending on the relative intensity of features, ‘spottiness’ as a result of noise amplification becomes visible after ~100 iterations in the darker part of the image and after ~1000 iterations in the brighter parts. (TIFF) [file pone.0130717.s009.tiff]

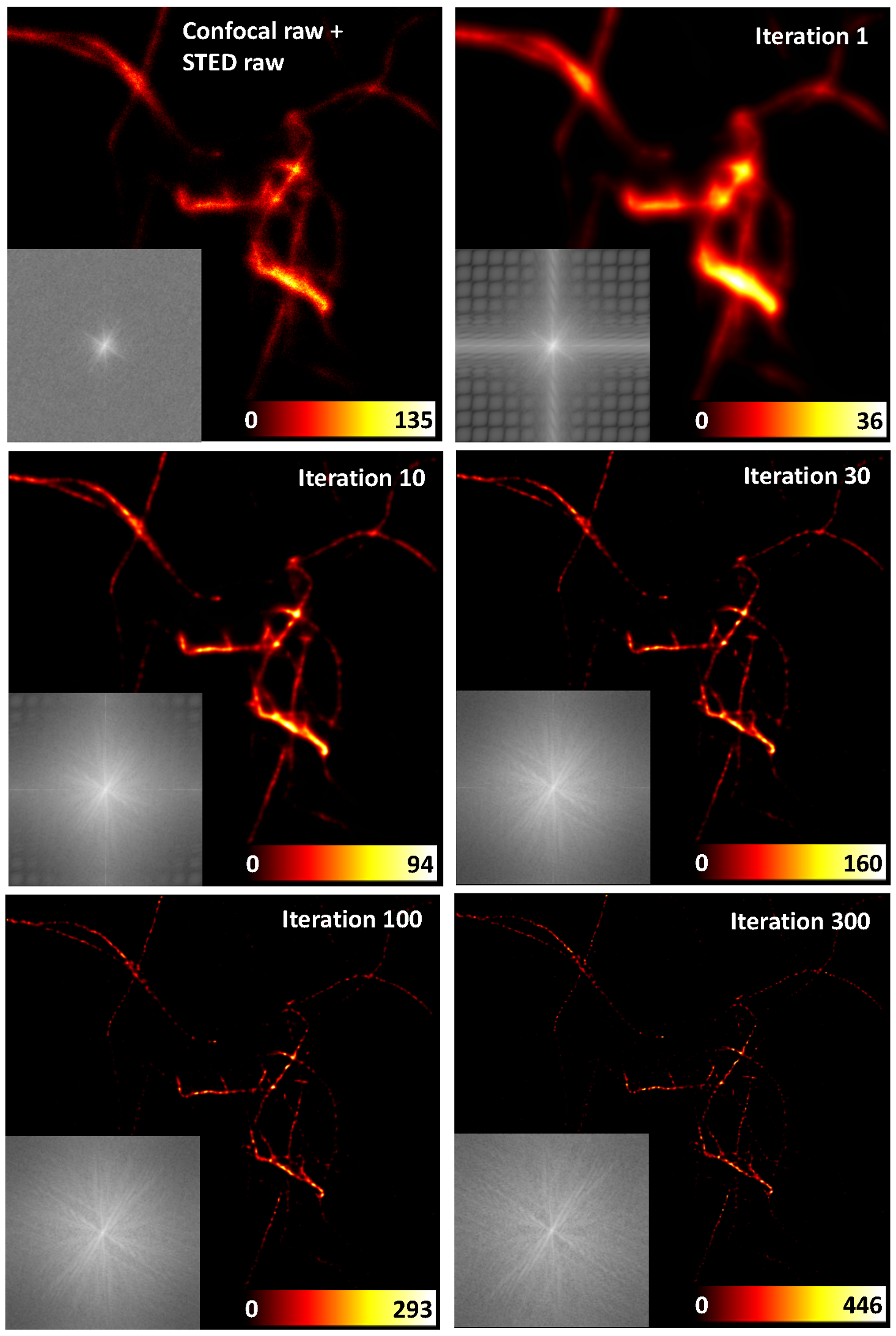

Supplement: S9 Fig — The sum of the raw STED image (counts in the third time gate) and the raw confocal image (counts in the first time gate) is shown together with the power spectrum of the discrete Fourier transform (inset), see Fig 4 (left column). Joint deconvolution of the raw images was performed as described by the application of the RL algorithm using a Lorentzian PSF with a FWHM diameter of 70 nm and a Gaussian PSF with a FWHM diameter of 300 nm. Reconstructed images after 1, 10, 30, 100, and 300 iterations are shown. Higher frequencies are recovered faster (less iterations) than in the case of confocal deconvolution only (S8 Fig). Compared to the STED-only deconvolution (S7 Fig), more iterations are required to reconstruct the high frequencies in the image. For a detailed comparison of the joint multi-image deconvolution with the deconvolution of the STED image alone, see S3 Fig. (TIFF) [file pone.0130717.s010.tiff]

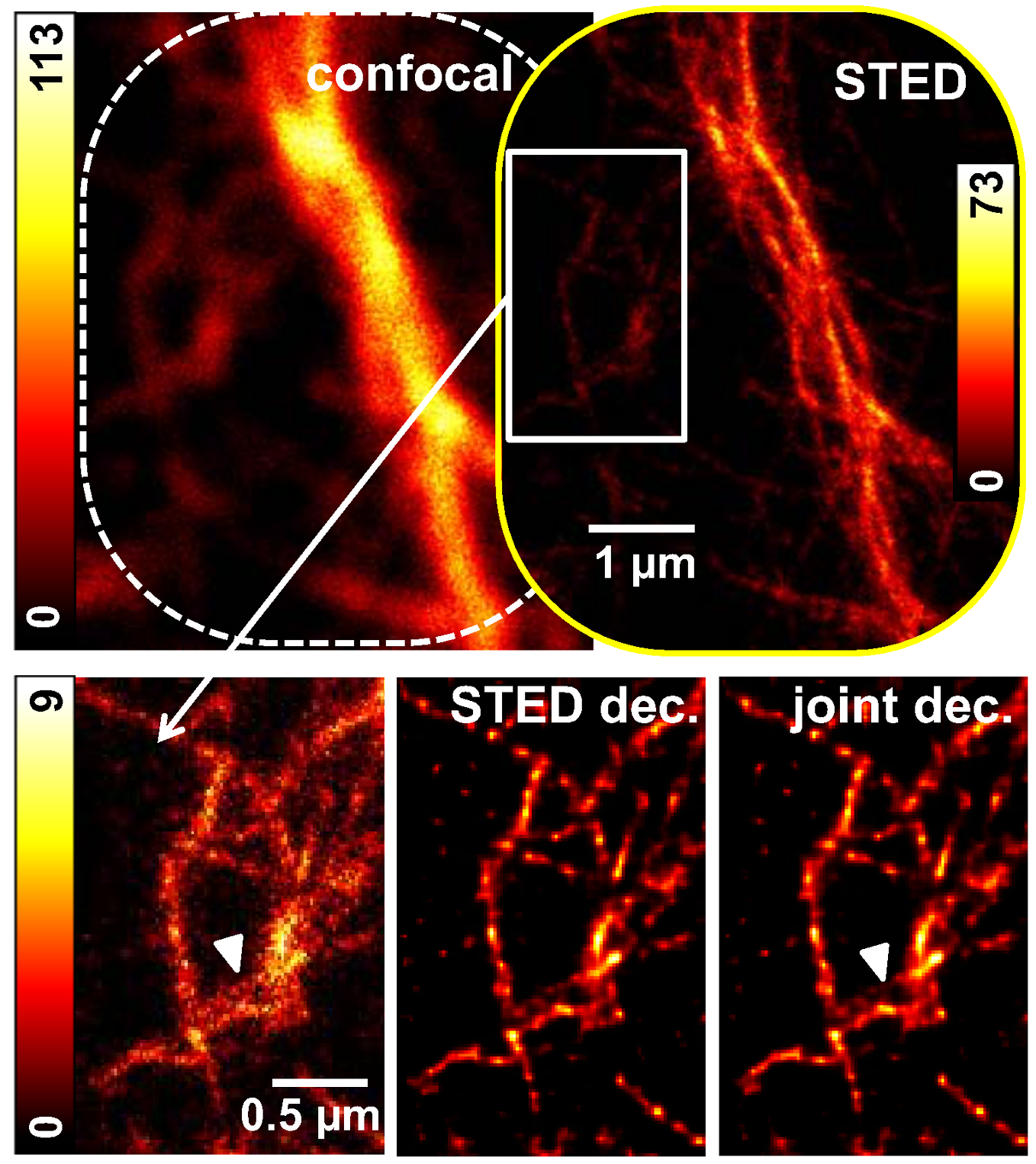

Supplement: S10 Fig — (A) Diffraction-limited and-unlimited images of a gated STED acquisition of a filament structure attached to the glass cover slip and labeled with the dye Atto647N. STED power of 5 mW at 2.5 MHz repetition rate. In the bottom row, the marked region of the STED image is shown enlarged and compared to the results of a Richardson-Lucy deconvolution of the STED image (STED dec.), and the result of a joint deconvolution (joint dec.). At the position marked by the white triangle two adjacent filaments may be assumed in the raw image, while in the jointly deconvolved image this feature appears suppressed stronger than in the case of the conventional deconvolution. This demonstrates that through the relative reduction of background and noise in the deconvolved image also badly defined features at low brightness may be suppressed. Parameter for the conventional deconvolution: 12 iterations with a Lorentzian functions of width 60 nm (determined from Fig 5C); 49 Iterations with Lorentzian function (60 nm) and Gaussian (300 nm) weighting input images by 1:1. (TIFF) [file pone.0130717.s011.tiff]

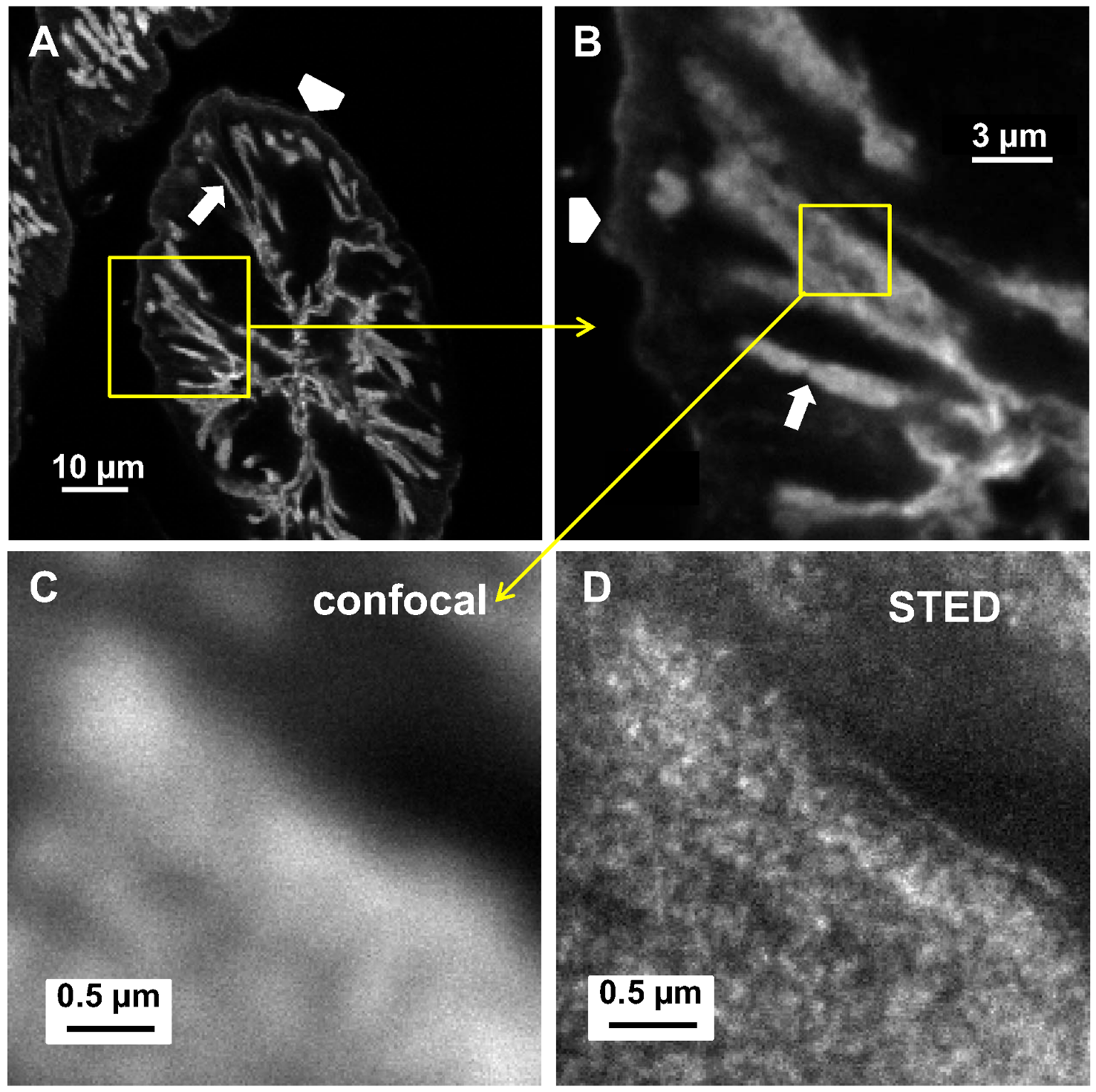

Supplement: S11 Fig — Gland cryosections (10 µm thick) were labeled with the F-actin probe Abberior STAR635-phalloidin. (A-C) A confocal overview image as well as enlarged views of regions indicated as yellow rectangles are shown. The basal region of the secretory cells showed weak fluorescence (thick arrow) indicating only marginal infoldings compared to the apical membrane [72,73]. In contrast, the canaliculi appeared as brightly fluorescent structures with widths in the micrometer range, indicating F-actin filament bundles lining the canaliculi (thin arrow). (D) STED image of the region shown in (C). TEM studies have unraveled further infolding of the canaliculi forming densely packed sheet like microvilli (so-called microplicae) with a microplicae having a thickness of 60–80 nm, while exhibiting variable lengths and widths. In addition, the microplicae are closely packed forming a fine extracellular space of approx. 10–20 nm between two adjacent microplicae [69]. Thus, this microplicae arrangement could represent an appropriable target for STED microscopy. However, no substructures of the canaliculi could be observed in confocal images (B-C). In the corresponding STED image (D) substructures within the canaliculus could be assumed, which were blurred out in the corresponding confocal image in (C). Nevertheless, the dense microplicae arrangement and thus the high density of F-actin seems to be hindering STED imaging, especially with diffraction-limited resolution in z-direction. The dense F-actin packing most probably influences the labeling efficiency, complicating the access of dye molecules to different parts of the microplicae. Thus, the labeled structure could differ from the defacto F-actin filament bundles, resulting in a heterogeneous fluorescence signal distribution as can be seen in (D). (TIFF) [file pone.0130717.s012.tiff]
